# *Pseudomonas aeruginosa* Biofilm Formation and Persistence, along with the Production of Quorum Sensing-Dependent Virulence Factors, Are Disrupted by a Triterpenoid Coumarate Ester Isolated from *Dalbergia trichocarpa*, a Tropical Legume

**DOI:** 10.1371/journal.pone.0132791

**Published:** 2015-07-17

**Authors:** Tsiry Rasamiravaka, Olivier M. Vandeputte, Laurent Pottier, Joelle Huet, Christian Rabemanantsoa, Martin Kiendrebeogo, Abel Andriantsimahavandy, Andry Rasamindrakotroka, Caroline Stévigny, Pierre Duez, Mondher El Jaziri

**Affiliations:** 1 Laboratoire de Biotechnologie Végétale, Université Libre de Bruxelles, Gosselies, Belgium; 2 Laboratoire de Formation et de Recherche en Biologie Médicale, Faculté de Médecine, Université d'Antananarivo, Antananarivo, Madagascar; 3 Laboratoire de Pharmacognosie, de Bromatologie et de Nutrition Humaine, Université Libre de Bruxelles, Brussels, Belgium; 4 Laboratoire de Biodiversité et de Biotechnologie, Institut Malgache de Recherches Appliquées, Antananarivo, Madagascar; 5 Laboratoire de Biochimie et de Chimie Appliquées, Université de Ouagadougou, Ouagadougou, Burkina Faso; 6 Service de Chimie Thérapeutique et de Pharmacognosie, Université de Mons, Mons, Belgium; UC Berkeley, UNITED STATES

## Abstract

Recently, extracts of *Dalbergia trichocarpa *bark have been shown to disrupt *P*. *aeruginosa *PAO1 quorum sensing (QS) mechanisms, which are key regulators of virulence factor expression and implicated in biofilm formation. One of the active compounds has been isolated and identified as oleanolic aldehyde coumarate (OALC), a novel bioactive compound that inhibits the formation of *P*. *aeruginosa* PAO1 biofilm and its maintenance as well as the expression of the *las* and *rhl* QS systems. Consequently, the production of QS-controlled virulence factors including, rhamnolipids, pyocyanin, elastase and extracellular polysaccharides as well as twitching and swarming motilities is reduced. Native acylhomoserine lactones (AHLs) production is inhibited by OALC but exogenous supply of AHLs does not restore the production of virulence factors by OALC-treated cultures, indicating that OALC exerts its effect beyond AHLs synthesis in the QS pathways. Further experiments provided a significant inhibition of the global virulence factor activator *gacA* by OALC. OALC disorganizes established biofilm structure and improves the bactericidal activity of tobramycin against biofilm-encapsulated PAO1 cells. Finally, a significant reduction of *Caenorhabditis elegans* paralysis was recorded when the worms were infected with OALC-pre-treated *P*. *aeruginosa*. Taken together, these results show that triterpenoid coumarate esters are suitable chemical backbones to target *P*. *aeruginosa* virulence mechanisms.

## Introduction

Over the last decades, pathogenic bacteria have presented an increasing multi-drug resistance prevalence all over the world [1, 2], a situation that has stimulated the search for new potential antibacterial drug targets [3, 4]. Inhibiting the bacterial virulence without killing the pathogen is an attractive anti-pathogenic approach that is increasingly explored [5, 6] with the putative advantage to generate less selective pressure as compared to antibiotics [7]. Quorum sensing (QS), a bacterial cell-to-cell communication, is used by many bacteria to detect their critical cell density by producing and perceiving diffusible signal molecules in order to coordinate a common behavior such as the expression and regulation of virulence factors, motility and biofilm formation [8, 9]. Therefore, the inhibition of QS systems might be a more valuable approach than targeting a single particular virulence factor for therapeutic or prophylactic control of infections.

The opportunistic pathogen *P*. *aeruginosa* is known to be an important human, animal and plant pathogen that produces several virulence factors. Its QS systems are probably the best-characterized among Gram-negative bacteria [10]. *P*. *aeruginosa* possesses two main QS systems (*las* and *rhl*) which drive the production (by the synthetases LasI and RhlI) and the detection (by the transcription factors LasR and RhlR) of the acylhomoserine lactones (AHL) *N*-(3-oxododecanoyl)-L-homoserine lactone (3-oxo-C12-HSL) and *N*-butanoyl-L-homoserine lactone (C4-HSL), respectively [11]. The *las* system controls LasB elastase, LasA protease, Apr alkaline protease, and exotoxin A [12] while the *rhl* system enhances the production of rhamnolipids, pyocyanin, LasB elastase, hydrogen cyanide, and cytotoxic lectins which are all involved in cellular toxicity and acute infection [13, 14]. The *las* and the *rhl* systems are organized in a hierarchical manner such that the *las* system regulates the *rhl* system at the transcriptional and post-transcriptional levels [15, 16]. In addition, *P*. *aeruginosa* releases a third intercellular signal, 2-heptyl-hydroxy-4-quinolone (designated the *Pseudomonas* quinolone signal [PQS]), which interacts with the acylhomoserine lactones (AHLs) systems in an intricate way [17]. The PQS system is incorporated into the QS hierarchy in times of cell stress, and acts as a link between the *las* and *rhl* quorum-sensing systems [18]. In this QS regulatory cascade, the *las* and *rhl* systems are positively influenced by the global activator GacA and the global regulator Vfr at both the transcriptional and post-transcriptional levels [19, 20].

Biofilms are surface-associated communities enclosed within an extracellular matrix [21] mainly composed of polysaccharides, proteins, nucleic acids, lipids and other macromolecules and chemicals [22]. Particularly, extracellular polysaccharides are a crucial component of the matrix, and carry out a range of functions such as promoting attachment to surfaces and other cells, building and maintaining biofilm structure, as well as protecting the cells against environmental assaults and predation, including antimicrobials and host defenses [23, 24].

QS systems and biofilm formation are crucial components in the development of acute and chronic infections, particularly for *P*. *aeruginosa* [25, 26], in which they play important roles in bacterial persistence and reduce sensitivity to antimicrobials [27]. Accordingly, molecules that affect the regulation of both QS mechanisms and biofilm formation could be powerful allies for conventional antibiotics in the struggle against bacterial infections [28, 29].

Recently, we demonstrated that a crude extract of *Dalbergia trichocarpa* bark inhibits the production of QS-dependent virulence factors in *P*. *aeruginosa* (pyocyanin, protease and elastase) as well as biofilm formation [30]. The present study describes the isolation, the identification and the biological characterization of the major bioactive compound in *D*. *trichocarpa* extract that inhibit both QS and biofilm formation without affecting bacterial growth.

## Materials and Methods

### Plant material

Stem bark samples of *D*. *trichocarpa* Baker. were collected in Madagascar from trees growing close to the city of Morondava (near the Kirindy forest, with the following GPS coordinates: 20° 04.120’ S 44° 39.250’ E, elevation 88 m). Collection authorization was delivered by MINENVEF (Ministry of Environment and Forest) authority represented by the Direction of Conservation, Biodiversity and Protected Area of Madagascar. Collection was carried out outside the protected Kirindy forest (tree cutting permission number 183/13/MEF/SG/DGF/DCB/SAP/SCB from 09/01/2013). Voucher specimen were deposited at FOFIFA (Centre National de la Recherche Appliquée au Développement Rural, BP 1690 Ampandrianomby 101 Antananarivo, Madagascar) and PBZT herbarium (Botanic and Zoologic Park of Tsimbazaza, Rue Fernand KASANGA Tsimbazaza 101 Antananarivo, Madagascar). *D*. *trichocarpa* Baker. is in the “Lower Risk/Least Concern” category according to the IUCN Red list of threatened Species (http://www.iucnredlist.org/details/36223/0).

### Bacterial strains, plasmids, and culture conditions

PAO1 strain and its derivatives were grown (37°C, agitation 175 rpm) in LB-MOPS broth (50 mM, pH 7) supplemented with carbenicillin (300 μg mL^-1^) when appropriate. Plasmids (S1 Table) were used and introduced in PAO1 as previously described [31].

*P*. *aeruginosa* PAO1 mutant strains were obtained from the Transposon Mutant Collection (Department of Genome Sciences, University of Washington; http://www.gs.washington.edu/labs/manoil/libraryindex.htm) and include mutants 11174 (ΔPA1432, Δ*lasI*), 17281 (ΔPA1430, Δ*lasR*), 32454 (ΔPA3476, Δ*rhlI*) and 3452 (ΔPA3477, Δ*rhlR*) [32]. When required, the medium was supplemented with 10 μM (final) 3-oxo-C12-HSL or C4-HSL as described previously [33].

### Chemicals

Naringenin, naringin and 4-nitro-pyridine-*N*-oxide (4-NPO) were purchased from Sigma-Aldrich and dissolved in 100% DMSO. Antimicrobial drugs (azithromycin and tobramycin) were purchased from TCI (Tokyo chemical industry Co. LTD, Japan) and dissolved in deionized water. The AHLs 3-oxo-C12-HSL and C4-HSL were purchased from Sigma-Aldrich and dissolved in 100% DMSO.

### Chromatographic purification of the major bioactive compound from *D*. *trichocarpa* barks

Dried powdered samples of *D*. *trichocarpa* stem barks (10 kg) were macerated overnight at room temperature in 20 L *n*-hexane and extracted with 60 L *n*-hexane by percolation (1 liter for 1 h). The gathered *n*-hexane extracts were filtered on Whatman paper, evaporated under vacuum and the resulting residue (40 g) was stored at -20°C. The residue was dissolved in 30 mL of *n*-hexane, loaded onto a chromatography column (35 by 4 cm) filled with silica gel 60 F_254_ (63–200 μm / 70–230 mesh; Merck) and eluted with *n*-hexane and a step gradient of ethyl acetate (100:10 to 10:100) to afford 6 fractions (fractions F1 to F6) and then with ethyl acetate and a step gradient of methanol (100:10 to 10:100) to afford 3 fractions (fractions F7 to F9). The fractions were evaporated and stored at -20°C. The fraction most inhibitory of QS and biofilm was further fractionated by preparative HPLC using RP C18 column (Altima HP, 13 by 250 mm; 5μm) that was eluted with a gradient of water-acetonitrile (10:90 in 3 min, 10:90 to 0:100 in 4 min, 0:100 in 2 min, 0:100 to 10:90 in 1 min, 10:90 in 5 min). Subfractions were collected, monitoring at 300 nm, using Buchi fraction collector C-660 with SepacoreRecord software assistance, and evaporated for anti-QS/anti-biofilm activities. The active subfraction was further purified on prepHPLC with the same conditions until positive peak purity, as assessed by spectroscopy (diode array detection under ChemStation 9.0 software) and TLC (Silicagel RF_254_ Merck; mobile phase, Hexane-ethyl acetate [70–30]; detection: UV illumination (254 and 366 nm) and after developing by spraying with 10% (v/v) H_2_SO_4_ followed by heating at 110°C for 10 min).

### Electrospray ionization mass spectrometry (ESI-MS) analysis and NMR spectroscopy of purified compound

The purified active compound (OALC) was analyzed by LC-ESI-MS with direct infusion into a Finnigan LCQ DUO mass spectrometer. The ESI parameters were as follows: solvent acetonitrile; concentration loaded 10 μg mL^-1^; negative ionization mode; nebulizer tip set at 250°C and 4.52 kV; cone voltage set at 5 kV; sheath gas (nitrogen) flow rate at 28 arbitrary units; collision energy at 70 eV; MS data were acquired in the *m/z* range from 50 to 2000. ^1^H- and ^13^C-NMR, COSY, NOESY, HSQC, and HMBC spectra were recorded on a Bruker Avance 400 NMR spectrometer. Standard pulse sequences and parameters were used for the NMR experiments and all chemical shifts were reported in parts per million (ppm, δ).

### Gene expression and β-Galactosidase measurements

To monitor gene expression of QS-dependent (*lasB* and *rhlA*), QS-regulatory (*lasR/I* and *rhlR/I*), *gacA* and *vfr* genes, the *β*-galactosidase activity induced by reporter genes was measured using *o*-nitrophenyl-*β*-D-galactopyranoside [34, 35]. After growth in liquid LB-MOPS-Carbenicillin at 37°C and 175 rpm for 18 hours, PAO1 reporter strains were washed twice in fresh LB medium and resuspended in liquid LB-MOPS-Carbenicillin. PAO1 reporter strains inoculums (50 μl) were incubated (37°C with 175 r.p.m. agitation) for 18 hours in 1 mL LB-MOPS-Carbenicillin (initial A_600nm_ of culture comprised between 0.020 and 0.025) supplemented with 10 μl of OALC dissolved in DMSO (200 μM) or 10 μl of DMSO (1%, v/v). Additionally, the flavanone naringenin, known as QS quencher [33], and its inactive glycoside (naringin) were used as positive and negative controls, respectively. After incubation, the bacterial density was assessed by spectrophotometry (A_600nm_) and the gene expression by the *β*-galactosidase assay.

For global gene transcription monitoring, a QS-independent gene, i.e. the isocitrate lyase-encoding *aceA* gene expression was also evaluated [36].

### Rhamnolipids extraction and quantification

For rhamnolipids extraction, *P*. *aeruginosa* PAO1 was grown at 37°C with agitation at 175 rpm for 18 h in 1 mL LB-MOPS medium supplemented with OALC (200 μM), naringin (4 mM) or naringenin (4 mM) or DMSO (1%, v/v). Bacterial cultures were centrifuged (3200 × g, room temperature, 5 min) and 1 mL of cell-free supernatant (Filtre Ultrafree-CL filters Millipore) was mixed with 1 mL of ethyl acetate and vigorously vortexed. Phase separation was then realized by centrifugation in a tabletop centrifuge at maximum speed (1 min). The upper, rhamnolipid-containing phase was transferred to a new reaction tube. This procedure was repeated three times. Finally, the organic solvent was removed by evaporation in a vacuum centrifuge.

Rhamnolipids were quantified by a methylene-blue-based method as described by Pinzon and Ju, [37] with some modification. Briefly, the rhamnolipid extract of each sample was dissolved in 4 mL of chloroform and put in contact with a freshly prepared methylene blue solution (200 μL of a 1 g l^-1^ methylene blue and 4.9 mL of distilled water). The pH of this methylene blue aqueous solution is adjusted to 8.6 ± 0.2 (usually achieved by adding 15 μL of a 50 mM borax buffer). After being vigorously mixed for 4 minutes, the samples were left to stand for 15 min for complexation. One mL of the chloroform phase was transferred in a new Eppendorf tube and mixed by vortexing with 500 μl of HCl 0.2 N. Finally, 200 μl of the superior acid phase, containing a portion of the complexed methylene blue, was transferred in a 96-well microplate (Greiner clear) and the absorbance was measured at 638 nm with SpectraMax M2 device (Molecular Devices) against an HCl 0.2 N blank. The absorbance values were converted to rhamnolipids concentration using a calibration curve established by applying the same procedure to standard rhamnolipids solutions of different concentrations.

### Homoserine lactone quantification

AHLs (C4-HSL and 3-oxo-C12-HSL) were extracted from PAO1 cultures and quantified by LC-ESI-MS as proposed by Makemson et al. [38]. *P*. *aeruginosa* PAO1 was grown at 37°C with agitation at 175 rpm for 18 h in 5 mL LB-MOPS medium supplemented with OALC (200μM), naringenin (4 mM) or naringin (4 mM) or DMSO (1%, v/v). Bacterial cultures were centrifuged (3220 × g, room temperature, 5 min) and supernatants (4 mL) were acidified with 80 μl glacial acetic acid prior to being extracted three times with ethyl acetate (4 mL). Ethyl acetate extracts were combined, evaporated to dryness and dissolved in 1 mL acidified ethyl acetate (0.1%, v/v, glacial acetic acid). For background measurement, supernatants (4 mL) were alkalinized with 80 μl 4 M NaOH before ethyl acetate extraction, hydrolyzed lactone rings being too polar to be extracted. ESI-MS quantification was done by direct infusion into a Finnigan LCQ DUO mass spectrometer under soft ionization conditions (positive ionization mode; nebulizer tip set at 250°C and 4.52 kV; cone voltage set at 5 kV; sheath gas (nitrogen) flow rate: 30 arbitrary units) that did not fragment the AHLs. Scans were averaged over 1 min. The peak intensities for C4-HSL (pseudomolecular ion, m/z = 172; ammonium adduct, m/z = 189; sodium adduct, m/z = 194; solvent adduct, m/z = 260) and 3-oxo-C12-HSL (pseudomolecular ion, m/z = 298; ammonium adduct, m/z = 315; sodium adduct, m/z = 320; solvent adduct, m/z = 386) were combined and converted to concentrations by using a standard curve generated from the pure compounds. Background readings from hydrolyzed samples extracted with ethyl acetate were subtracted from those of the acid-extracted bacterial cultures before conversion. To evaluate an eventual chemical-modulation activity of OALC, LB-MOPS medium supplemented with OALC and/or exogenous AHLs (3-oxo-C-12HSL and C4-HSL) were incubated for 18h and AHLs have been extracted following the same procedure.

### Quantitative analysis of pyocyanin and elastase production by *P*. *aeruginosa* PAO1

The production of pyocyanin and elastase was assessed according to previously described procedures [39, 40]. *P*. *aeruginosa* PAO1 or mutant strains were grown for 18 h in liquid LB-MOPS (supplemented with 60 μg mL^-1^ tetracycline for mutant strains). PAO1 cell suspension (50 μl) was added to 1 mL of LB-MOPS (starting A_600nm_ ranged between 0.02 and 0.025) supplemented with 10 μl of OALC dissolved in DMSO (200 μM) or 10 μl of DMSO (1%, v/v) or naringenin (4 mM) or naringin (4 mM). When required, the medium was supplemented with 10 μM of 3-oxo-C12-HSL or C4-HSL as described previously [33]. After 18 h of growth, samples were taken to assess growth (A_600nm_) and pyocyanin production. The LasB elastase production was assessed through the measurement of elastase activity using elastin-Congo Red [40].

### Assessment of bacterial growth

Relative growth of *P*. *aeruginosa* PAO1 after 18 hours in the presence of OALC was evaluated by measuring the cell density at A_600nm_ with a SpectraMax M2 device (Molecular Devices). The effect of OALC on *P*. *aeruginosa* PAO1 growth kinetic was evaluated by evaluating PAO1 cell density (A_600nm_) over 22 hours culture, confirmed by cell counting (colony-forming units, C.F.U) at times 8 and 18 h.

### Motility assays

The swarming motility was examined using LB agar plate described previously [41]. Briefly, LB agar (0.6%) medium supplemented with glutamate (0.05%) and glucose (0.2%) was autoclaved for sterilization. Sterilized medium was cooled at temperature between 45–50°C and then supplemented (DMSO 1% or OALC at 200 μM or naringenin at 4 mM or naringin at 4 mM) before being poured into compartmented Petri dishes. Gelled plates were inoculated at their center with 5 μl of a bacterial culture of PAO1 cells diluted to a turbidity of 1 at 600 nm and placed at 37°C when inoculums spots are dry. Bacteria spreading from the inoculation spot were measured after 24 hours. The twitching motility was assayed as previously described [42] with slight modification. Briefly, plates with LB agar (1%) supplemented (DMSO 1% or OALC at 200 μM or naringenin at 4mM or naringin at 4mM) poured to an average depth of 3 mm were prepared and dried briefly. A hole (about 1mm diameter) was dug in the center of each compartment through the agar and 5 μl of *P*. *aeruginosa* PAO1 cells diluted to a turbidity of 1 at 600 nm were spotted inside the hole, dried briefly and incubated at 37°C for 48 hours. After the incubation period, bacteria spreading between the agar and the bottom of the petri dish were measured. For diameters measurement, the agar was discarded from petri dish and twitching motility zones were easily visualized by staining for 1 minute with 0.1% (w/v) of crystal violet as proposed by Darzins [43].

### Biofilm quantification and synergistic activity with tobramycin

*P*. *aeruginosa* PAO1 was grown overnight in LB medium at 37°C with agitation. After growth, the culture of *P*. *aeruginosa* PAO1 was diluted with Biofilm Broth (BB) medium as described by Khalilzadeh et al. [44] and 25 μl of the diluted culture was added to 470 μl of BB medium (initial A_600nm_ of culture comprised between 0.14 and 0.16) supplemented with 5 μl of DMSO (1%, v/v) or OALC (200 μM) or naringenin (4mM) or naringin (4mM). Planktonic bacteria were transferred in sterile tube and assessed for cell counting (colony-forming units, C.F.U). Adherent biofilms were washed three times with water (2mL) and fixed with 2 mL of methanol (99%). After 15 min, the methanol was discarded, and the plates were dried at room temperature. Crystal violet (0.1% in water) was then added to each well (2 mL/well), and the plates were incubated for 30 min at room temperature. Crystal violet was then discarded, and stained biofilms were washed three times with 1 mL of water. Acetic acid (33% in water) was added to the stained biofilms (2 mL) in order to solubilize the crystal violet, and the absorbance of the solution was measured at 590 nm with a SpectraMax M2 device (Molecular Devices).

The biofilm formation by PAO1 cells was also examined in glass coverslips cultures by fluorescence microscopy. Two distinct assays were adopted in order to assess the effects of compounds in biofilm development and in one-day-old biofilm. In both cases, the bactericidal activities of tobramycin in one-day-old biofilm-encapsulated PAO1 cells was also assessed. Tobramycin was chosen because it has been shown that QS inhibition greatly enhances the sensitivity of *P*. *aeruginosa* to this antibiotic and increases clearance of *P*. *aeruginosa* in a foreign-body infection model [28, 45]. First assay follows the same culture conditions as described above. After 24 h incubation, tobramycin (100 μg mL^-1^) was added to 1-day-old treated biofilms. The biofilm development and bacterial viability in biofilms were assessed using the LIVE/DEAD *bac*light bacterial viability kit (Invitrogen, Molecular probes). The growth medium was removed and replaced by 500 mL of a solution of SYTO 9 and propidium iodide diluted 400 fold in BB medium. Biofilms were incubated for 15 min and PAO1 cells were examined using a Leica DM IRE2 inverted fluorescence microscope coupled to a CCD camera (Leica DC350 FX) and equipped with FITC and Texas red filters. To estimate the % viability of biofilm-encapsulated bacteria for each treatment, the glass coverslip was submerged in 2 mL of PBS solution and sonicated (WVR Ultrasonic cleaner, HF45KHz, 80W) for 1 min in order to unbind the biofilm. The collected biofilm suspension was then assessed for viability using LIVE/DEAD *bac*light bacterial viability kit (Invitrogen, Molecular probes) following fluorescence microplate reader protocols as described by the manufacturer. The integrated intensities of the green (530 nm) and red (630 nm) emission of suspensions excited at 485 nm were acquired using SpectraMax M2 device, and the green/red fluorescence ratios (Ratio G/R) were calculated and reported to the linear curve obtained from the relationship between % live bacteria and Ratio G/R of biofilm-encapsulated PAO1 cells grown without tobramycin.

For the second assay, PAO1 cells were grown statically in BB medium for 24 hours at 37°C in 24-well polystyrene plates to form biofilm. Tested molecules as described above and/or tobramycin (100 μg mL^-1^) were added and incubated for a further 24 hours and the biofilm development and bacterial viability in biofilms were assessed as described for the first assay.

### Total extracellular polysaccharides and alginate quantification

For extracellular polysaccharides extraction, the method using ethanol was followed as described by Gong et al. [46]. Briefly, *P*. *aeruginosa* PAO1 was grown at 37°C with agitation at 175 rpm for 18 h in 5 mL LB-MOPS medium supplemented with OALC (200 μM), naringin or naringenin (4 mM) or DMSO (1%, v/v). The bacterial culture was centrifuged (3200 × g, room temperature, 5 min) and the supernatant discarded. The freshly harvested cell pellet was resuspended in 10 mL 0.22% formaldehyde (ACS grade, Fisher Scientific) in 8.5% sodium chloride for 2 h in a 4°C incubator. Following the exposure to formaldehyde, the suspension was centrifuged (3200 × g, 4°C, 15 min) and the resulting pellet containing the polysaccharides was resuspended in 10 mL deionized (DI) water (Millipore, Milli-Q Academia). Then the suspension was centrifuged again (3200 × g, 4°C, 15 min) to rinse away any remaining cellular material, the pellet was collected, weighted, resuspended in DI water (50 μL per mg of pellet), sonicated for 3 min (460 = H Elma Transsonic Lab-Line Instruments, Germany) and centrifuged once more at 3200 × g, 4°C for 15 min (Eppendorf Centrifuge 5810R). This polysaccharides pellet was suspended in 5 mL 10^−2^ M KCl and 10 mL pure and cold ethanol, incubated overnight at 4°C to reprecipitate the polysaccharides that were centrifuged (3200 × g, 4°C, 20 min). After decantation, the purified pellet was suspended in 10 mL DI water and the total amount of carbohydrates was evaluated with the Phenol-Sulfuric Acid (PSA) method [47]. Two mL of this polysaccharides solution were added with 50 μL of a 80% phenol solution (Fisher Scientific) and, carefully, 5 mL of 95% sulfuric acid (Fisher Scientific), incubated, 30 min at 25°C, 4 h at room temperature (22–25°C) and measured at 480 nm with SpectraMax M2 device (Molecular Devices) referring to glucose as a standard [48].

Alginate was quantified by a slightly modified carbazole-based method that detects uronic acids [49]. One mL overnight grown *P*. *aeruginosa* culture was mixed with 1 mL of 0.85% NaCl and the A_600_ of the solution was determined after 1 min vortex. After elimination of bacteria (13 000 x *g*, 60 min), the alginate present in 1 mL of the supernatant was precipitated by addition of 1 mL 2% cetyl pyridinium chloride and collected (13 000 × *g*, 20 min). The pellet was dissolved in 1 M NaCl, precipitated again with 1 mL of cold (-20°C) isopropanol, centrifuged (13 000 *g*, 20 min, 4°C), resuspended in 1 mL saline and stored at 4°C. Fifty μl of serial dilutions of standard (sodium alginate 0.1–1 mg mL^-1^) or sample were added with 200 μl of 25 mM sodium tetraborate in concentrated sulfuric acid, heated for 10 min at 100°C, cooled at room temperature for 15 min and carefully added with 50 μl of 0.125% carbazole in absolute ethanol. After heating at 100°C for 10 min and cooling at room temperature for 15 min, 300 μl of the reaction product were transferred into an appropriate cuvette and measured at a wavelength of 550 nm.

### *C*. *elegans* paralytic killing and toxicity assay

Viability of adult nematodes deposited on the lawn of PAO1 was assessed as described previously with some modification [50, 51]. *P*. *aeruginosa* PAO1 (virulent strain), ΔPA1430, (Δ*lasR*) and ΔPA3477 (Δ*rhlR*) (reduced virulent strains) were grown overnight in LB and then diluted 100-fold into fresh broth. Brain Heart Infusion agar plates containing either DMSO 1%, OALC (200 μM), naringenin (4 mM), naringin (4 mM) or 4-NPO (100 μM) were spread with 400 μl of diluted culture and then incubated at 37°C for 24 h to form lawns of bacteria. Synchronized culture L4 nematodes or adult wild-type nematodes (From University of Minnesota), obtained as previously described [52], were washed off stock plates and suspended in a minimal volume of PBS buffer (pH 6.5). One hundred μl of nematodes suspension were placed onto the *P*. *aeruginosa* lawns and incubated at 20°C; the droplets dried within 30 min and deposited the nematodes on the lawn. Likewise, nematodes free of *P*. *aeruginosa* were deposited on plates containing OALC or naringenin or DMSO to evaluate an eventual toxicity. Plates were then sealed with parafilm and incubated at 20°C. After 4 h, deposited nematodes were retrieved with PBS buffer (pH 6.5) and the obtained worm suspension was transferred into a 15-mL tube and centrifuged (1300 rpm, 2 min), rinsed twice with 5 mL PBS and resuspended in 1 mL PBS for preparing the fluorescence revelation of dead worms as described elsewhere [53]. Briefly, washed-worm suspension was labelled by adding 200 μl of a 5(6)-carboxyfluorescein diacetate (CFDA) working solution and leaving for 30 min at room temperature in the dark. After centrifugation (1300 rpm, 2 min) and washing with 5mL PBS to eliminate the excess of fluoresceine, the pellet was resuspended in 1 mL PBS and divided into 200 μl aliquots containing around 20–40 worms. The proportion of dead worms was then measured by visual counting (triplicate counting) in fluorescence microscopy.

### Statistics

All experiments were performed in quintuplicate and repeated in three independent assays. The data were statistically analyzed by conducting Student’s *t* tests and a *p* value ≤ 0.01 was considered as being significant.

## Results

### Oleanolic aldehyde coumarate isolated from *D*. *trichocarpa* bark extract reduces the expression of QS-regulated *lasB* and *rhlA* genes

**Fig 1:**
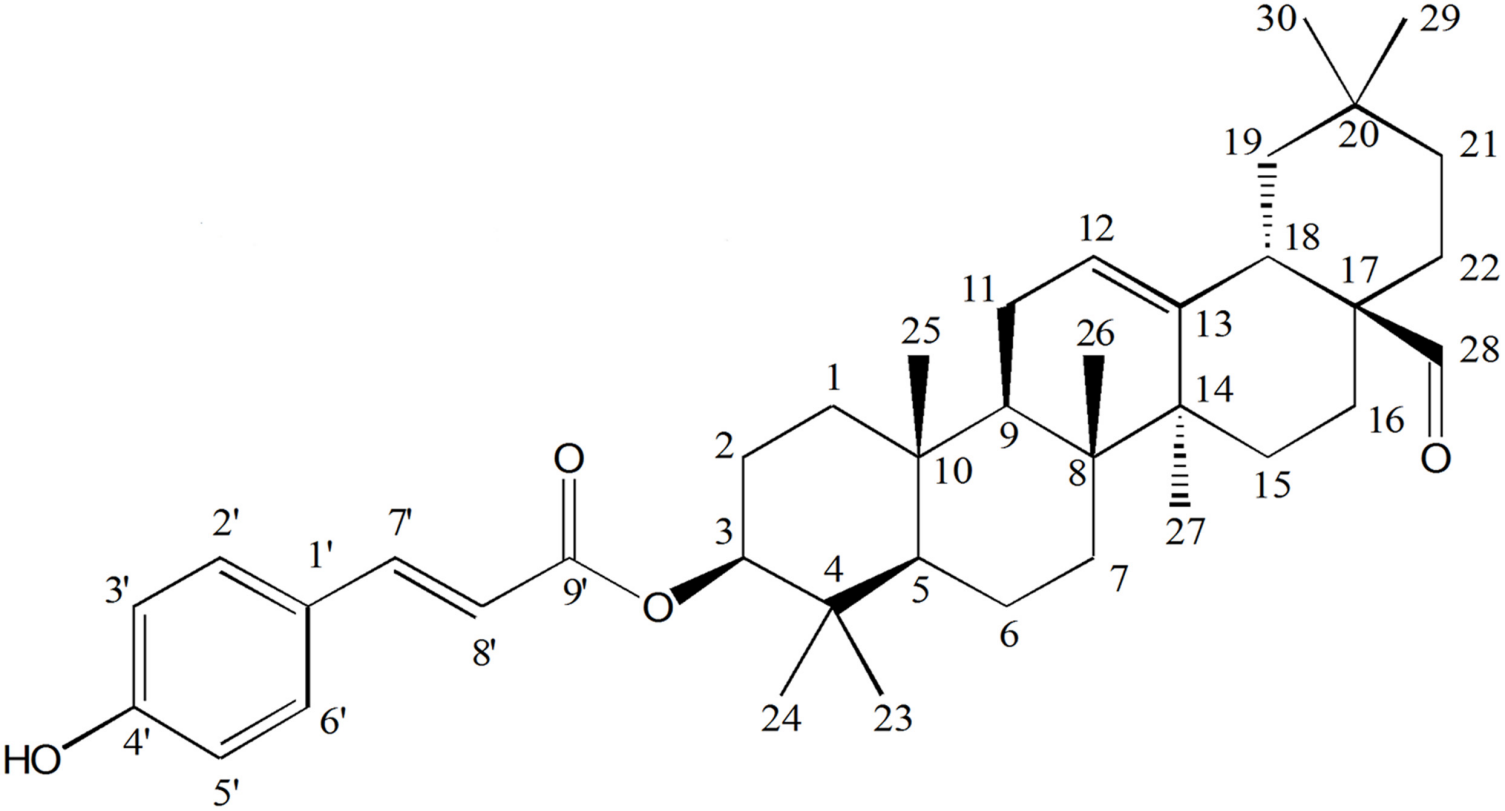
Chemical structure of the bioactive compound assigned as 3β-hydroxyolean-12-en-28-al 3-*p*-coumarate (oleanolic aldehyde coumarate, OALC).

To identify the QS inhibitory (QSI) compound(s) occurring in the *D*. *trichocarpa* bark (DTB) extract, the chromatographic fractionation was bio-guided by quantification of *lasB* and *rhlA* gene expression as well as biofilm formation in *P*. *aeruginosa* PAO1. The procedures and results that led to the isolation of the active compound are detailed in S1 Text and S1 Fig. The chemical structure of the isolated active compound was elucidated through spectroscopic analyzes, including UV, mass and NMR spectral data which are detailed in S2 Text, S2 and S3 Figs, leading to its identification as a 3β-hydroxyolean-12-en-28-al 3-*p*-coumarate or oleanolic aldehyde coumarate (OALC, MW: 586.52, C_39_H_54_O_4_; Fig 1 and S2 Table).

The effect of OALC on *lasB* and *rhlA* expression was then evaluated at different concentrations (from 50 to 200 μM). As shown in S4A and S4B Fig, significant reduction of the expression of both genes is observed from 150 μM of OALC. For instance, at 200 μM of OALC 61 ± 5% of inhibition for *lasB* and 69 ± 5% of inhibition for *rhlA* was recorded. To monitor the effect of OALC on global gene transcription, a QS-independent gene, i.e. the isocitrate lyase-encoding *aceA* gene expression was evaluated (see experimental procedures for details). As shown in S4C Fig, *aceA* expression as well as bacterial growth were not affected by OALC at the tested concentrations. Consequently, as more than 50% inhibition in QS gene expression was recorded in presence of 200 μM of OALC, this concentration was used in all further experiments. Higher concentrations were not assessed due to limited quantity of purified OALC.

To evaluate the impact of OALC on *P*. *aeruginosa* PAO1 growth and viability, growth kinetics and C.F.U. measurements were recorded in the presence of 200 μM of OALC. As shown in S5 Fig, *P*. *aeruginosa* growth kinetics and viability were not significantly affected by OALC as compared to the DMSO treatment; this was shown by both culture turbidity measurements (S5A Fig) and C.F.U. quantifications after 8 and 18 hours of growth (S5B and S5C Fig). This result suggests that the decrease in QS gene expression and biofilm formation were not due to a drop in cell viability.

### OALC reduces the production of QS-dependent virulence factors (pyocyanin, elastase and rhamnolipids) as well as native AHLs production in *P*. *aeruginosa* PAO1

**Fig 2:**
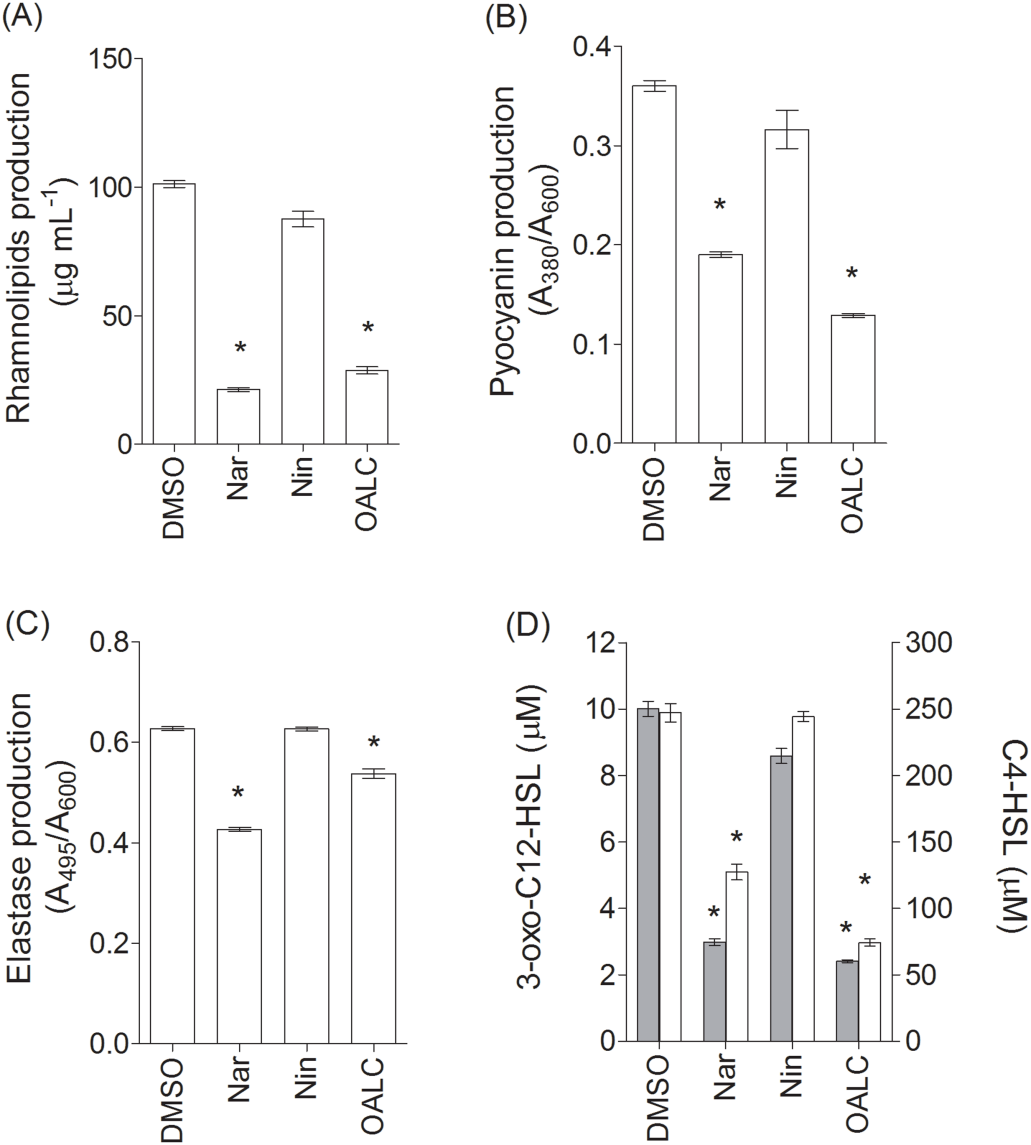
Effect of OALC on virulence factors and acylhomoserine lactones production in *P*. *aeruginosa* PAO1. (A) rhamnolipids production, (B) pyocyanin production, (C) Elastase production. The cell density of the bacteria was assessed at 600 nm and elastase production was assessed via an elastolysis assay and calculated as the ratio between *A*_495_ and *A*_600_. The rhamnolipids production was measured using methylene-blue-based method and expressed in μg mL^-1^. Pyocyanin was extracted, quantified by absorbance measurements at 380 nm and calculated as the ratio between *A*_380_ and *A*_600_. (D) Quantification of *N*-(3-oxododecanoyl)-L-homoserine lactone (3-oxo-C12-HSL; grey bar) and *N*-butanoyl-L-homoserine lactone (C4-HSL; clear bar) produced by PAO1 cells. Acylhomoserine lactones were extracted and quantified by mass spectrometry. Error bars represent the standard errors of the means and all experiments were performed in quintuplicate with three independent assays and asterisks indicate samples that are significantly different from the DMSO controls (Student’s *t*-tests; *P* ≤ 0.01). Naringenin (Nar) and naringin (Nin) were used as a quorum sensing inhibitor control and negative control, respectively.

As *lasB* and *rhlA* expressions are reduced in PAO1 cells treated with OALC, this could be reflected in the production of associated-virulence factors. Thus, we assessed the effect of OALC (at 200 μM) on the production of three QS-dependent virulence factors, i.e. rhamnolipids, pyocyanin and elastase. As shown in Fig 2, a drastic inhibition of the production of rhamnolipids (75 ± 4% of inhibition; Fig 2A) and pyocyanin (64 ± 3% of inhibition; Fig 2B) was observed when PAO1 strains were grown in the presence of OALC compared to PAO1 strains grown with DMSO. Additionally, OALC also reduced elastase production (19 ± 4% of inhibition; Fig 2C) and OALC did not degrade elastine-Congo red in bacteria-free tests (data not shown).

The expression of the *las*- and *rhl*-dependent virulence factor genes is positively controlled by the transcription factor complexes LasR/3-oxo-C-12-HSL and RhlR/C4-HSL, respectively [9]. Thus, the observed reduction in the expression of *lasB* and *rhlA* genes induced by OALC could be linked to a reduction of 3-oxo-C12-HSL and C4-HSL levels in culture media. As shown in Fig 2D, OALC significantly reduced the concentrations of both auto-inducers after 18 h of growth, by 3- and 4-fold for C4-HSL and 3-oxo-C12-HSL, respectively. To address the matter of whether OALC reacts directly with the AHL released in culture medium, LB-MOPS medium supplemented with OALC and/or exogenous AHLs (3-oxo-C-12HSL and C4-HSL) have been incubated for 18h and AHLs were then extracted and quantified (see experimental procedures for details). No significant difference in extracted AHLs was observed, suggesting the absence of reactivity towards AHLs (data not shown). At this stage, reduction of native AHLs in culture media is presumably linked to a direct or indirect inhibition of AHLs production by OALC.

To address the matter of whether AHLs supply could restore the production of QS-virulence factors in the presence of OALC, 3-oxo-C12-HSL or C4-HSL were added exogenously to OALC-treated PAO1 cells. As shown in S6 Fig, the addition of 3-oxo-C12-HSL or C4-HSL at 10 μM (see experimental procedures for details) did not restore the production of pyocyanin or elastase in OALC-treated wild-type PAO1 cells (S6A and S6B Fig). The same experiment was performed with the ΔPA1432 and ΔPA3476 mutant strains (which lack functional *lasI* and *rhlI* synthetase genes, respectively) to avoid interference with native AHLs (S6C Fig). The exogenous addition of 3-oxo-C12-HSL to the ΔPA1432 strain significantly increased elastase production, but adding OALC reduced this production to the base level (S6C Fig), confirming that an exogenous supply of AHLs is not sufficient to compensate the effect of OALC. As shown in S6D Fig, the ΔPA3476 mutant was unable to produce pyocyanin unless C4-HSL was exogenously supplied (while 3-oxo-C12-HSL had no effect). Similarly to Δ*lasI* strain, when OALC was added to C4-HSL-induced Δ*rhlI* cells, the level of pyocyanin production was three-fold lower than that observed when C4-HSL alone was added (S6D Fig). These data, consistent with those obtained on the wild type strain PAO1 supplemented with exogenous AHLs, confirm that the reduction of native AHLs induced by OALC is not compensated by exogenous AHLs supply. All together these data indicate that OALC could exert its effect beyond AHLs synthesis in the QS pathways. To further document this hypothesis, the expression of QS regulatory genes (*lasI/R* and *rhlI/R*) and two global activator genes (*gacA* and *vfr*) was investigated.

### OALC affects the expression of *lasI/R*, *rhlI/R* and the global activator *gacA*

**Fig 3:**
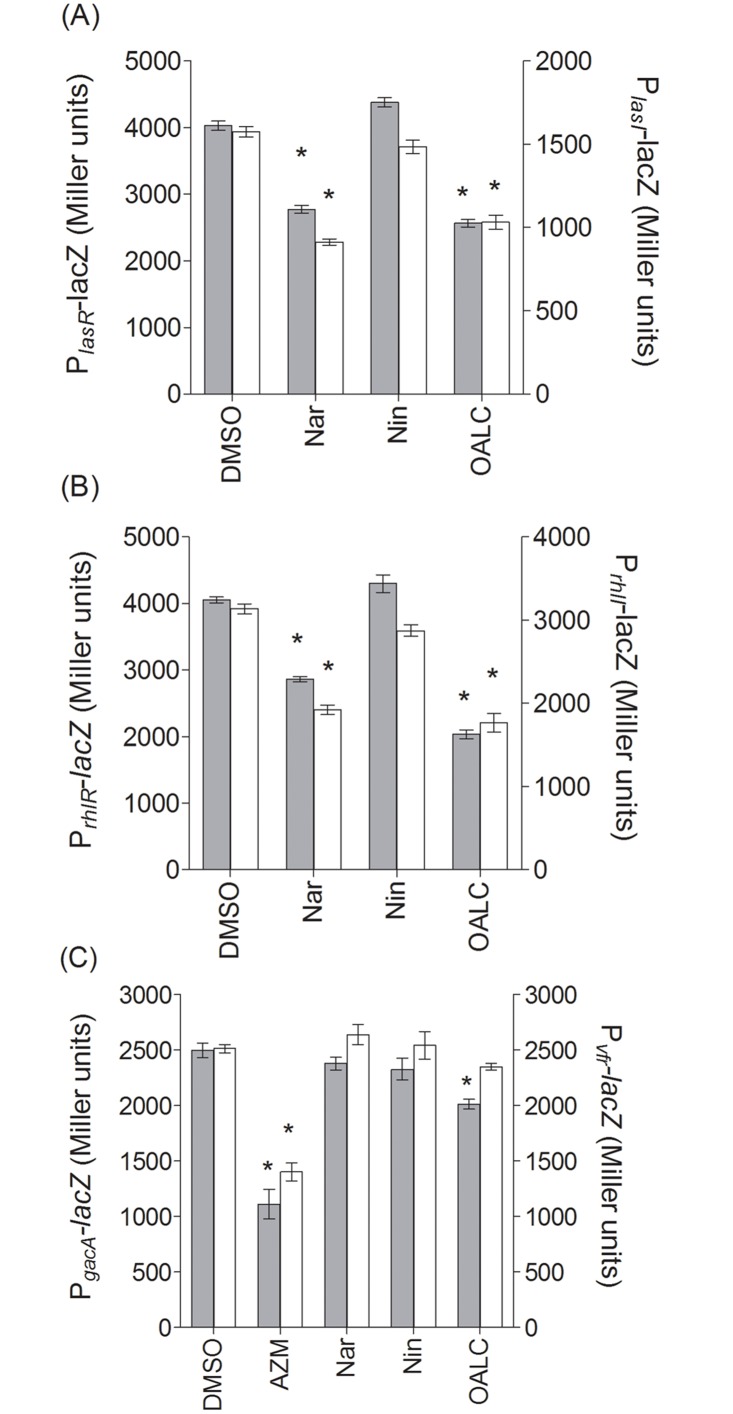
Effect of OALC on QS genes (*lasI/R and rhlI/R*) and global activator genes (*gacA* and *vfr*) expression in *P*. *aeruginosa* PAO1. (A) Effect of OALC on *lasR* (grey bar) and *lasI* (clear bar) expression following 18 hours of growth. (B) Effect of OALC on *rhlR* (grey bar) and *rhlI* (clear bar) expression following 18 hours of growth. (C) Effect of OALC on *gacA* (grey bar) and *vfr* (clear bar) expression following 18 hours of growth. Gene expression was measured as the β-galactosidase activity of the *lacZ* gene fusions and expressed in Miller units. Error bars represent the standard errors of the means; all experiments were performed in quintuplicate with three independent assays and asterisks indicate samples that are significantly different from the DMSO (Student’s *t*-tests; *P* ≤ 0.01).

As shown in Fig 3A and 3B, OALC significantly reduces (*p* ≤ 0.01) the expression of the *rhlI* and *lasI* genes (39 ± 3% and 33 ± 5% of inhibition, respectively). Similarly, OALC significantly reduces the expression of *lasR* and *rhlR* genes (35 ± 3% and 50 ± 3% of inhibition, respectively). In the hierarchical *P*. *aeruginosa*-QS cascade, the global activator GacA and Vfr are positioned upstream and exert a positive effect on the transcriptional regulators LasR and RhlR [19, 20]. In this respect, the effect of OALC on *gacA* and *vfr* genes expression was therefore investigated. As the effect of naringenin on the expression of the global activator genes *gacA* and *vfr* had not been previously evaluated, azithromycin (1 μg mL^-1^ final concentration) was preferred as positive inhibitory control [54]. As shown in Fig 3C, OALC exhibits a small but significant inhibitory effect on *gacA* expression (16 ± 4% of inhibition; *P* ≤ 0.01) but no effect on *vfr* expression.

### OALC affects biofilm formation in *P*. *aeruginosa* PAO1

**Fig 4:**
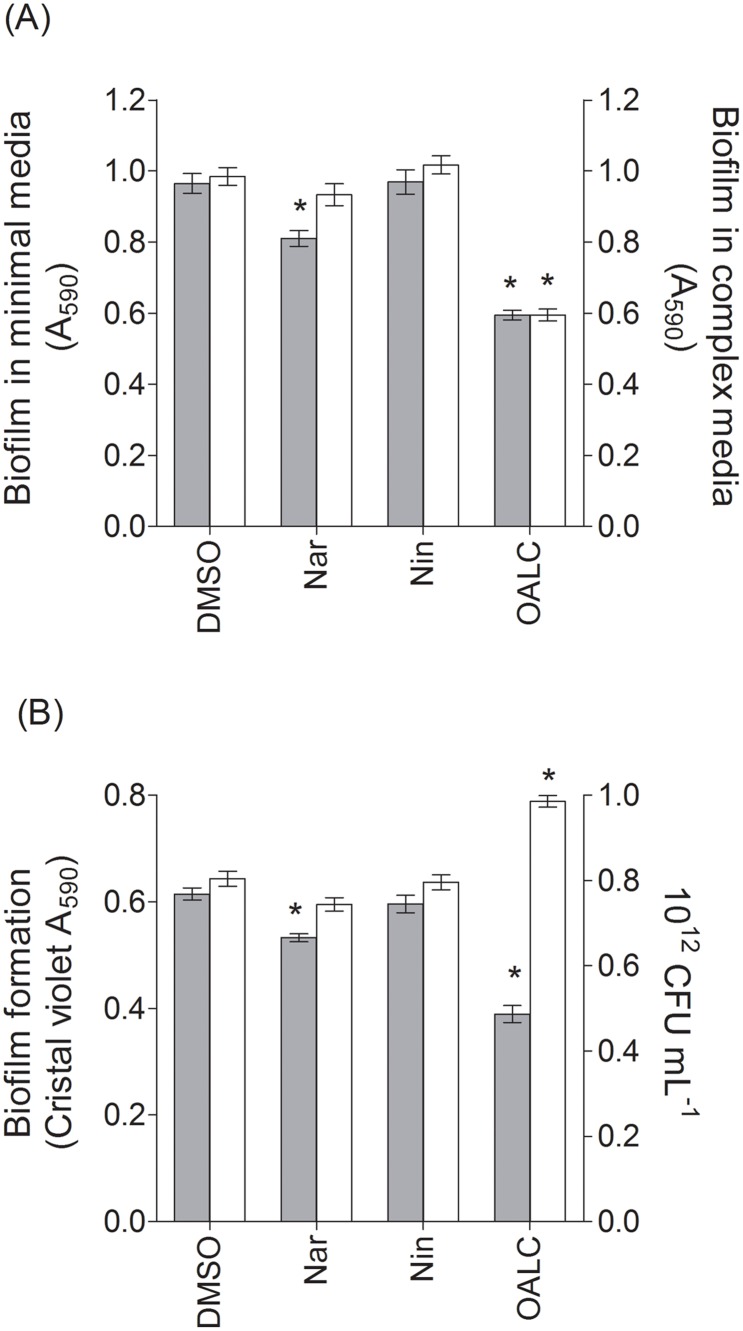
Effect of OALC on biofilm formation by *P*. *aeruginosa*. (A) Quantification of biofilm formation by *P*. *aeruginosa* PAO1 grown in minimal (grey bar) and complex media (clear bar) after static incubation at 37°C for 24 hours. (B) Quantification of biofilm formation by *P*. *aeruginosa* grown in in minimal media (grey bar) and C.F.U. measurement (clear bar) of planktonic bacteria after static incubation at 37°C for 24 hours. Biofilm formation was quantified by crystal violet staining and measured at A_590nm_. The cell density of the bacteria was assessed at 600 nm. Error bars represent the standard errors of the means; all experiments were performed in quintuplicate with three independent assays and asterisks indicate samples that are significantly different from the DMSO (Student’s *t*-tests; *P* ≤ 0.01).

As evidenced by the bio-guided chromatographic fractionation (see S1 Fig), OALC inhibits biofilm formation by *P*. *aeruginosa* PAO1. To further detail the effect of OALC on biofilm formation, its effect was compared in two growth media, a minimum and a complex medium (See experimental procedures) as biofilm development is nutritionally conditioned [55]. As shown in Fig 4A, OALC significantly reduced the biomass of the PAO1 biofilm in both minimal and complex media (44 ± 4% and 36 ± 5%, respectively). Additionally, in minimal media, a significant increase of planktonic bacteria is recorded compared to DMSO (Fig 4B), suggesting that OALC promotes planktonic bacteria lifestyle instead of biofilm.

**Fig 5:**
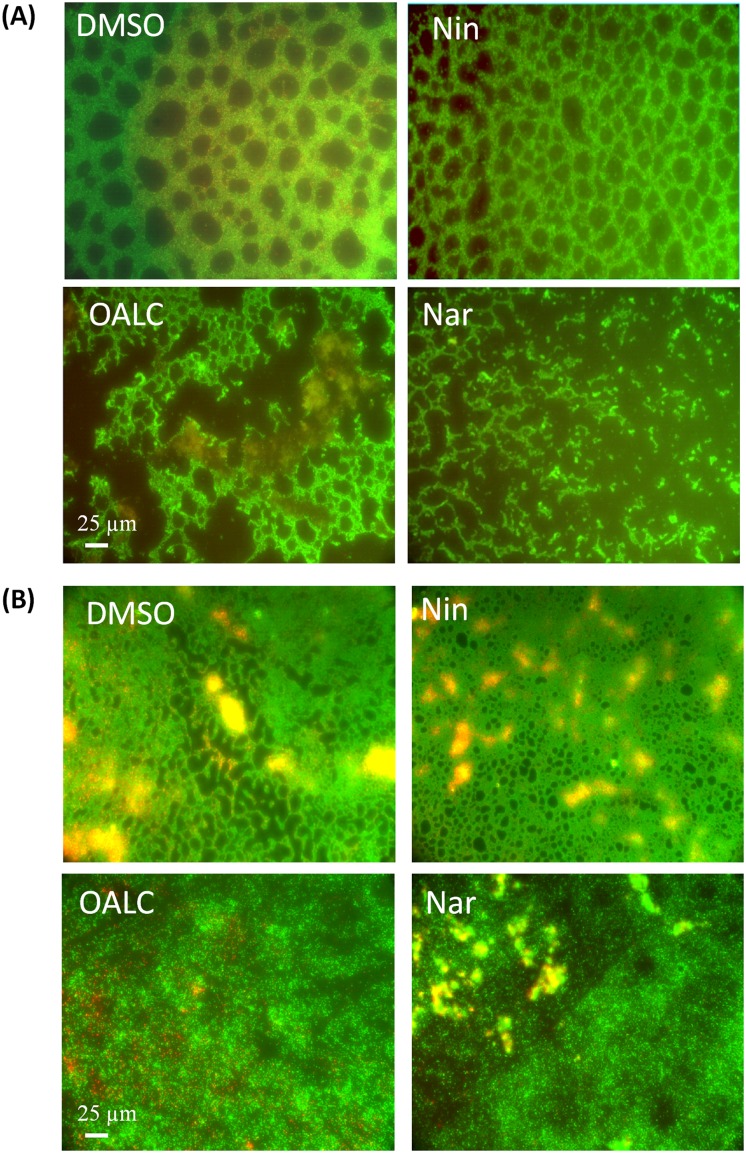
PAO1 biofilm phenotypes as affected by DMSO, naringin, naringenin or OALC. (A) Fluorescence microscopy images of PAO1 cells incubated statically at 37°C for 24 hours. Cells were visualized after staining with SYTO-9 (green fluorescence for living bacteria) and propidium iodide (red fluorescence for dead bacteria) furnished in the LIVE/DEAD *Bac*Light kit. (B) Fluorescence microscopic images of biofilm formation by PAO1 cells incubated for 24 hours and then treated for 24 hours with DMSO, naringin, naringenin or OALC. Fluorescence microscopy was achieved by using a Leica DM IRE2 inverted fluorescence microscope using a 40x objective lens and images were false-colored and assembled using Adobe Photoshop.

Visualization of *P*. *aeruginosa* biofilm by fluorescence microscopy using SYTO-9 and propridium iodide (to stain live and dead cells, respectively) indicated that PAO1 grown for 24 hours in static condition (and with DMSO) forms a thick and homogenous biofilm layer on coverslips with good cell-to-cell connections interspaced by uncolonized surfaces (Fig 5A). Similar biofilms were observed in cells treated with the negative control naringin, although confluent bacterial biofilms were visually less thick as observed with fluorescence microscopy. By contrast, OALC- and naringenin-treated PAO1 cells failed to establish compact cell-to-cell attachment resulting in a reduction of microcolonies confluence (Fig 5A). Interestingly, when applied to one day-old pre-formed biofilm, both OALC and naringenin disrupted the architecture of PAO1 biofilm, as shown by the presence of aggregates and less confluent microcolonies (Fig 5B). On the contrary, application of DMSO- and naringin on one-day-old biofilms had no impact (Fig 5). This result suggests that important components for PAO1 biofilm formation and structure maintenance are impaired in the presence of OALC.

### Major PAO1 phenotypes contributing to biofilm formation, including swarming, twitching motilities and extracellular polysaccharides, are affected by OALC

**Fig 6:**
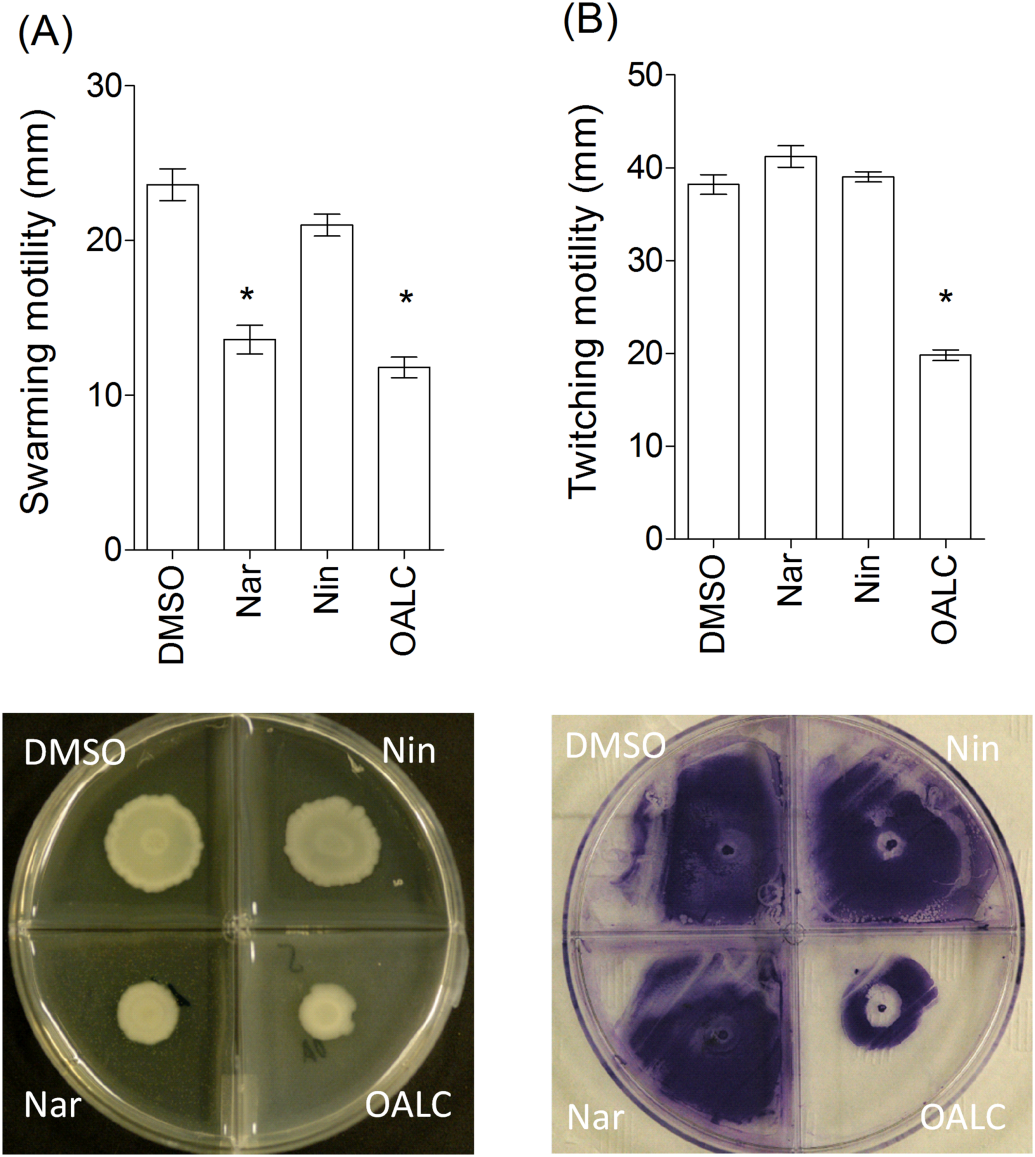
Effect of OALC on *P*. *aeruginosa* PAO1 motilities. (A) Swarming motility of *P*. *aeruginosa* PAO1 onto LB agar (0.6%) supplemented with glutamate (0.05%), glucose (0.2%) and DMSO (1%) or OALC (200μM final concentration), naringenin (Nar, 4 mM final concentration) or naringin (Nin, 4 mM final concentration). After incubation at 37°C for 24 hours, the zones of migration (down) from the point of inoculation were measured (up) for each condition. (B) Twitching motility of *P*. *aeruginosa* PAO1 onto LB agar (1%) supplemented with DMSO (1%), OALC (200μM final concentration), Nar (4 mM final concentration) or Nin (4 mM final concentration). The twitching zones were stained (down) and their diameters (up) measured after incubation at 37°C for 48 h. Error bars represent the standard errors of the means and all experiments were performed in quintuplicate with three independent assays and asterisks indicate samples that are significantly different from the DMSO (Student’s *t*-tests; *P* ≤ 0.01).

To invade surfaces, *P*. *aeruginosa* uses swarming and twitching motilities; both processes are under QS regulation and contribute to the initial stages of biofilm formation and architecture [56–58]. As shown in Fig 6, PAO1 swarming was significantly affected when grown onto LB agar supplemented with OALC or naringenin but not when DMSO and naringin were supplemented to the medium (Fig 6A). This reduction of bacterial motilities by OALC could contribute to reducing their ability to form confluent microcolonies (Fig 5A).

**Fig 7:**
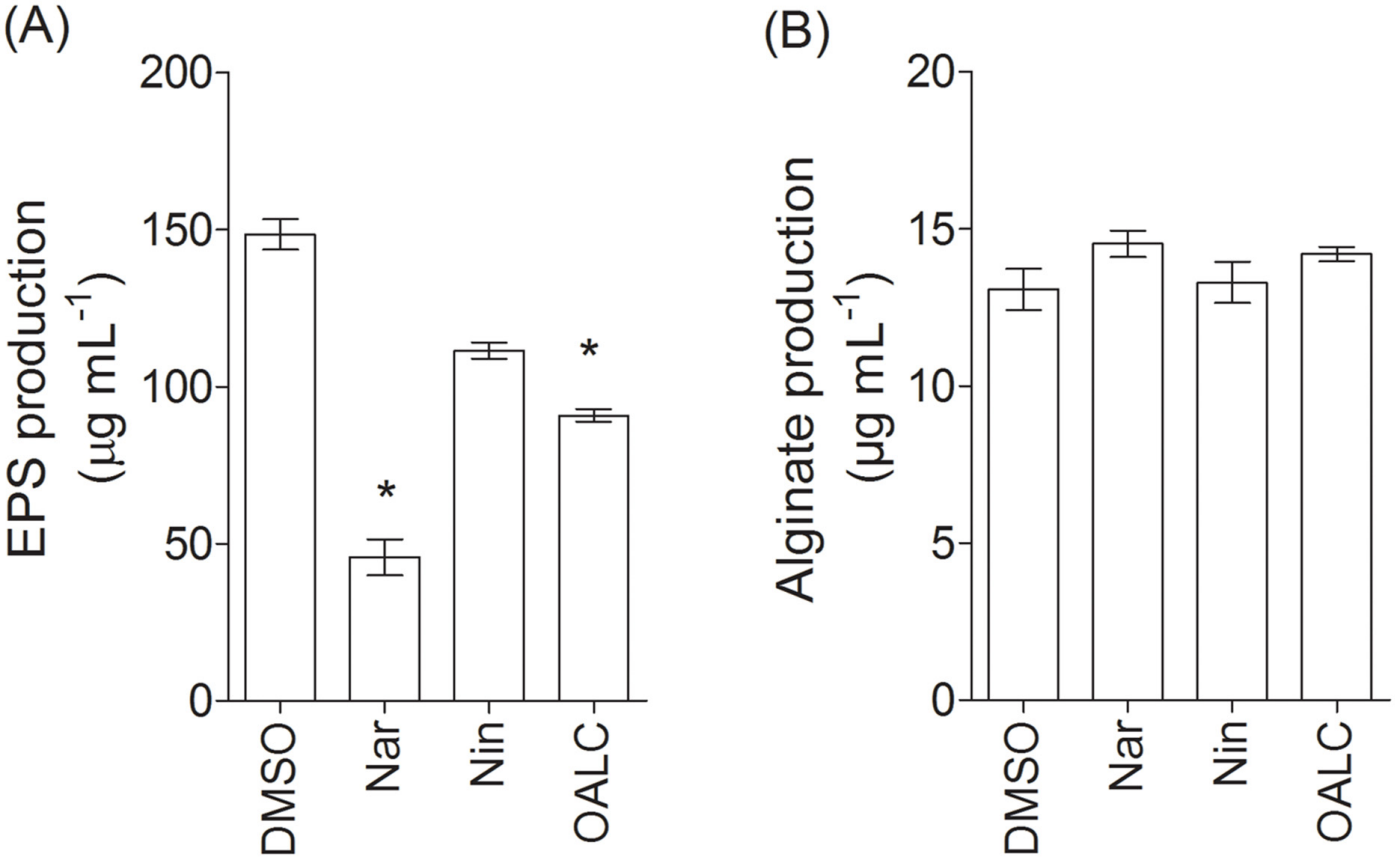
Effect of OALC on extracellular polysaccharides production by *P*. *aeruginosa* PAO1. (A) Quantification of total extracellular polysaccharides. The cell density of the bacteria was assessed at 600nm and extracellular polysaccharides production was measured using Phenol-Sulfuric Acid (PSA) method and expressed in μg mL^-1^ with glucose as standard. (B) Quantification of alginate. The cell density of the bacteria was assessed at 600nm and alginate production was measured using carbazole method and expressed in μg mL^-1^ with sodium alginate as standard. Error bars represent the standard errors of the means; all experiments were performed in quintuplicate with three independent assays and asterisks indicate samples that are significantly different from the DMSO (Student’s *t*-tests; *P* ≤ 0.01). Naringenin (Nar) and naringin (Nin) both at 4 mM final concentration were used as quorum sensing inhibitor positive and negative controls, respectively.

Fluorescence microscopy analyses revealed distinct micro-morphological alterations of the adherent bacteria and biofilm matrix after OALC and naringenin treatment, suggesting the disruption of matrix components (Fig 5). Consistently, the amount of extracellular polysaccharides produced by PAO1 after 24 hours of growth was reduced in OALC- and naringenin-treated PAO1 cells (40 ± 4% and 66 ± 5%, respectively) compared to the DMSO treatment (Fig 7A). However, the production of alginate, an acidic polysaccharide composed of non-repeating subunits of D-mannuronic and L-guluronic acid [59], was not significantly different compared to the DMSO treatment (Fig 7B). Thus, the global reduction of extracellular polysaccharides is presumably linked to reductions in Pel and/or Psl (polysaccharide synthesis locus) polysaccharides, a reduction that could negatively impact the biofilm structures of PAO1.

### OALC exhibits antibiotic-synergizing activities in biofilm

**Fig 8:**
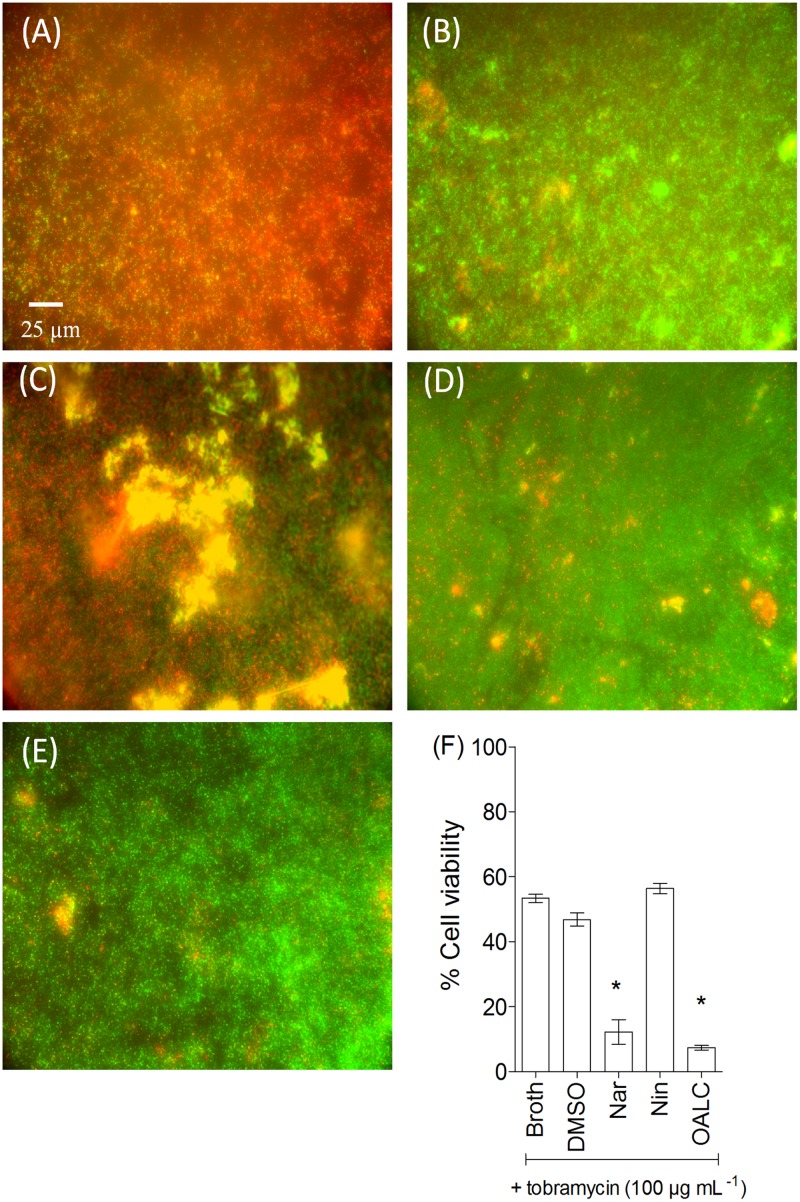
Synergistic activity of OALC with tobramycin against biofilm-encapsulated *P*. *aeruginosa* PAO1. PAO1 cells were incubated statically for 24 hours in the presence of DMSO, naringin (4 mM), naringenin (4 mM) or OALC (200 μM) and then treated for 24 hours with tobramycin (100 μg mL^-1^). (A) OALC + tobramycin, (B) tobramycin, (C) naringenin + tobramycin, (D) DMSO + tobramycin, (E) naringin + tobramycin. Assessment of bacterial viability and microscopy were performed as in Fig 6. (F) Quantification of bacterial viability. Error bars represent the standard errors of the means; all experiments were performed in quintuplicate with three independent assays and asterisks indicate samples that are significantly different from the DMSO (Student’s *t*-tests; *P* ≤ 0.01).

**Fig 9:**
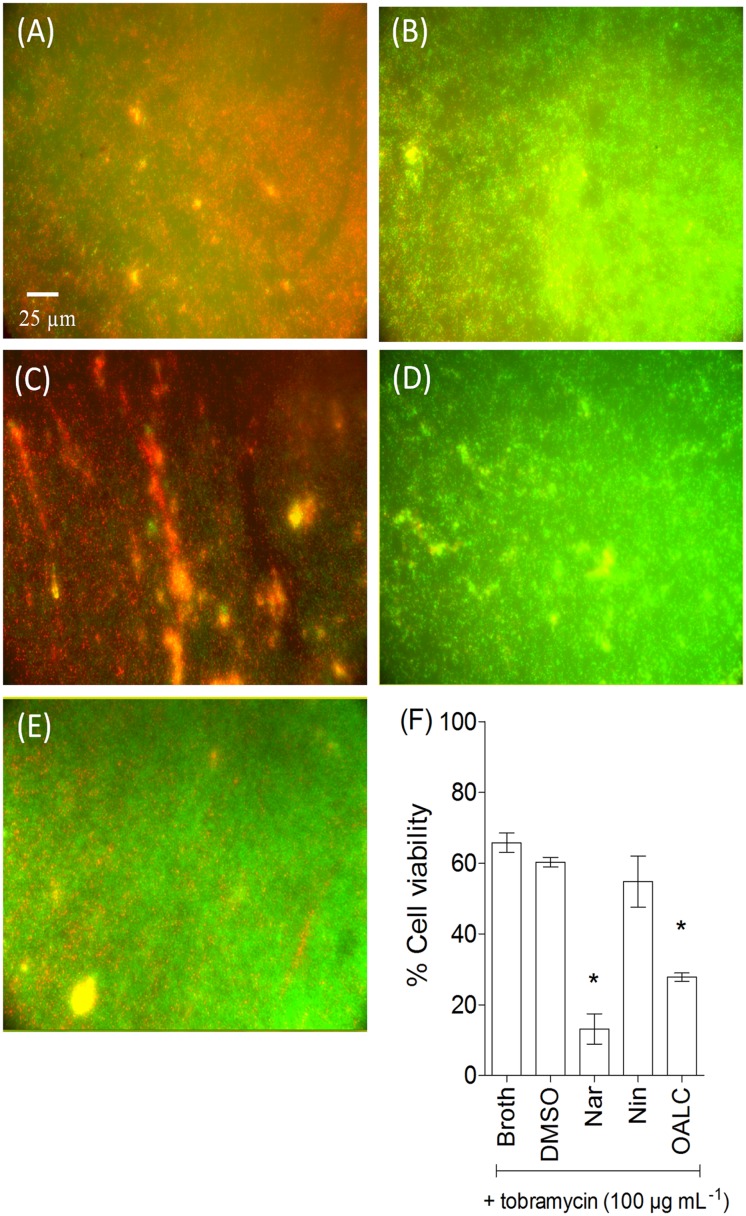
Synergistic activity of OALC with tobramycin against biofilm-encapsulated *P*. *aeruginosa* PAO1. PAO1 cells were incubated statically for 24 hours and then treated for 24 hours with tobramycin (100 μg mL^-1^) and DMSO, naringin (4 mM), naringenin (4 mM) or OALC (200 μM final concentration). (A) OALC + tobramycin, (B) tobramycin, (C) naringenin + tobramycin, (D) DMSO + tobramycin, (E) naringin + tobramycin. Assessment of bacterial viability and microscopy were performed as in Fig 6. (F) Quantification of bacterial viability. Error bars represent the standard errors of the means; all experiments were performed in quintuplicate with three independent assays and asterisks indicate samples that are significantly different from the DMSO (Student’s *t*-tests; *P* ≤ 0.01).

Since OALC affects both biofilm formation and structure maintenance (Fig 5) as well as EPS production (Fig 7), it is tempting to evaluate the protective ability of biofilm matrix treated with OALC against environmental aggressions such as antibiotics. For that, tobramycin has been selected because it is widely used to treat acute *P*. *aeruginosa* exacerbations in patients with cystic fibrosis [60] but appears less effective in biofilm-encapsulated *P*. *aeruginosa* compared to their planktonic counterparts [61]. Moreover, EPS has been demonstrated to be a barrier for antibiotic attacks by limiting the penetration of tobramycin [62, 63]. As shown in Fig 8, OALC in association with tobramycin significantly increased the effectiveness of the antibiotic against biofilm-encapsulated bacteria. Indeed, tobramycin introduced at 100 μg mL^-1^ in one day-old biofilm-encapsulated PAO1 cells was found to kill 90 ± 5% of OALC-treated biofilm cells (ie, OALC was introduced during the initial growth of PAO1 to form one day-old biofilm) (Fig 8A), whereas tobramycin alone could kill only 36 ± 4% (Fig 8B). Similar results were observed for the naringenin-tobramycin treatment (86 ± 5% of dead cells; Fig 8C), whereas the association of tobramycin with DMSO (Fig 8D) or naringin (Fig 8E) did not exhibit significant bactericidal effect (45 ± 5% and 40 ± 3%, respectively) compared to tobramycin alone. Although less efficient, this synergistic effect was also manifest when OALC-tobramycin are added to one day-old biofilm cultures (76 ± 3% of dead cells) contrarily to the associations tobramycin-DMSO and tobramycin-naringin (40 ± 4% and 44± 6%, respectively; Fig 9). These results suggest an improvement of antibiotic diffusion/penetration through the biofilm matrix in the presence of OALC which is correlated with the decrease of EPS production and biofilm architecture disruption induced by OALC.

### OALC reduces paralytic killing activity of *P*. *aeruginosa* PAO1 on *Caenorhabditis elegans*

*P*. *aeruginosa* PAO1 strains are known to cause death of *C*. *elegans* by neuromuscular paralysis [64], which has been demonstrated to be LasR-dependent [65]. Thus, the success of OALC in affecting *P*. *aeruginosa* PAO1 QS systems suggested that this molecule might also reduce *C*. *elegans* mortality in a PAO1-nematode model. Synchronized culture of wild type L4 adult nematodes obtained as described previously [52] (See experimental procedures for details) were therefore deposited on a lawn of PAO1 pre-treated with OALC, naringenin, naringin, DMSO or 4-NPO (a reference QSI agent) [50]. After 4 hours of incubation, dead worms were counted following fluorescence revelation as previously described [51, 53]. As shown in S7A Fig, more than 80% of the worms died within 4 hours onto plates containing PAO1 conditioned with DMSO 1%. When treated with 4-NPO (100 μM) or with OALC (200 μM), the wild-type PAO1 strain could kill only 27 ± 2% and 52 ± 2% of the worms, respectively. Similar results were observed with naringenin (52 ± 4% of dead worms). QS-defective strains Δ*lasR* and Δ*rhlR* pre-treated in the different conditions induce only 20 to 30% of nematode death after 4 hours (S7B and S7C Fig). However, naringenin at 4 mM and OALC at 300 μM (not 200μM) turned out to be toxic to *C*. *elegans*, with a death count of about 60 to 65% (S7D Fig).

## Discussion

Antimicrobial resistance is undoubtedly a growing worldwide public health threat so that the WHO foresees the emergence of a ‘post-antibiotic’ era during the 21st century in which common infections and minor injuries will have a dramatic impact on human death toll [2, 66]. Infection control using strategies inhibiting bacterial virulence and/or biofilm formation are increasingly explored as anti-pathogenic approaches because they are thought to minimize the selection pressure and the concomitant emergence of resistances experienced with strategies targeting basic cell mechanisms and leading to pathogen’s death [29, 67]. In the case of *P*. *aeruginosa*, several chemical structures (synthetic and natural) have been reported so far to present, at the same time, anti-QS, anti-biofilm and in-biofilm antibiotic-synergizing activities [68]. The hemisynthetic compound azithromycin (AZM, a macrolide antibiotic) was found to present both QS and biofilm inhibitory effects in *P*. *aeruginosa* when used at sub-bactericidal concentration [2.67 μM] [69]. A number of natural compounds have also been demonstrated to affect QS-controlled gene expression in *P*. *aeruginosa* and to reduce biofilm production; these include, among recently reported, 6-gingerol [at 10 μM] (a pungent oil of fresh ginger) [70], eugenol from clove [at 50 μM][71], ajoene [at 100 μM] (an allyl sulfide from garlic) [45], *S*-phenyl-L-cysteine sulfoxide [at 1 mM] [72] and some flavonoids (the flavan-3-ol catechin [at 4 mM] [35] from *Combretum albiflorum* and naringenin [at 4 mM] [33]). Herein, OALC exerts its activity at 200 μM in the range of other compounds reported elsewhere.

In the present report, the bio-guided fractionation of *D*. *trichocarpa* bark extract led to the isolation and identification of an oleanolic aldehyde coumarate (3β-hydroxyolean-12-en-28-al 3-*p*-coumarate or OALC) as a novel QSI and anti-biofilm compound without bactericidal effect. *D*. *trichocarpa* is traditionally used along the west coast of Madagascar as local medicine to treat various ailments such as laryngitis, diarrhea and rheumatic pains [73]. Besides, the antimicrobial activity of extracts from *Dalbergia* species including *D*. *parviflora* Roxb. and *D*. *horrida* Dennst. has also been reported and was mainly attributed to flavanone and isoflavone derivatives [74–76]. To the best of our knowledge, this is the first report of the occurrence of OALC in any genus/species of plant or other organisms. However, a closely related derivative, oleanolic acid coumarate, has been previously isolated from *Jatropha curcas* L. [77] and *Hippophae rhamnoides* L. [78] and was shown to present an antimycobacterial (*Mycobacterium tuberculosis*) activity [79]. Besides, Hu et al. [80] demonstrated that a closely related compound, 20β-hydroxyursolic acid coumarate, isolated from leaves of *Diospyros dendo* inhibits biofilm formation in *P*. *aeruginosa* PAO1; though, this compound was not screened for anti-QS activities.

In *P*. *aeruginosa* PAO1, OALC at 200 μM seems to display a large number of biological activities including QSI and anti-biofilm activities that implies multiple cellular targets. Indeed, OALC inhibits QS-regulatory (*lasI/R* and *rhlI/R*), QS-regulated (*lasB* and *rhlA*), global activator *gacA* genes expression (Fig 3) and alters biofilm formation and maintenance (Figs 4 and 5). Multiple studies demonstrate that QS systems contribute to the ability of bacteria to form biofilms [81, 82]; for instance, *P*. *aeruginosa* defective in QS are compromised in their ability to form biofilms [81, 83]. In the case of OALC, one can assume an indirect link between QS and biofilm formation through the control of essential components such as polysaccharides (Fig 7) and rhamnolipids (Fig 2) production, as well as swarming and twitching motility (Fig 6), that are all negatively regulated by OALC. Indeed, inactivation of the *rhlA* gene (crucial for rhamnolipids biosynthesis) causes an inhibition of swarming motility and reduces twitching motility [41, 84]. In addition, it has been reported that *P*. *aeruginosa* strains limited in swarming motility form biofilms containing non-confluent cell aggregates [58], a phenotype similar to that induced in OALC-treated PAO1 biofilm (Fig 5). In the same line, twitching motility, which has also been shown to be regulated by the *rhl* system [85], is necessary for the assembly of a *P*. *aeruginosa* cell monolayer into microcolonies [86]. Moreover, mutants deficient in rhamnolipids synthesis fail to maintain open channels, which are necessary for distribution of nutrients and oxygen within the biofilm matrix [87]. In addition, rhamnolipids are involved in microbial cell adhesion and biofilm development [88]. A reduction of rhamnolipids production as suggested by the down regulation of *rhlA* expression by OALC, might result in a decreased development or a disruption of developing biofilms. Thus, rhamnolipids reduction in OALC-treated PAO1 (Fig 2) as well as failure in swarming and twitching motilities (Fig 6) also contribute to explain the ability of OALC to disrupt preformed biofilm in *P*. *aeruginosa* (Fig 5). Similarly, it was recently shown that phenazine binding to extracellular DNA is important for *P*. *aeruginosa* biofilm formation (Das *et al*., 2015) [89]. Indeed, authors demonstrated significant decrease in biofilm formation by the *P*. *aeruginosa* Δ*phzA-G* mutant and enhancement in Δ*phzA-G* biofilms in the presence of exogenous pyocyanin. Since OALC reduces the production of pyocyanin (one of the phenazines produced by *P*. *aeruginosa*; Fig 2), biofilms might also be disrupted by a lack of pyocyanin in the extracellular matrix, thereby weakening the biofilm structure.

Exopolysaccharides are considered as crucial components for biofilm formation and its maintenance in *P*. *aeruginosa*. Indeed, most *Pseudomonas* strains, including PAO1 strain, rely on Psl and Pel polysaccharides for cell-surface, cell-to-cell interactions as well as for adherence to host cells [62] and for biofilm formation [23, 90, 91]. As total extracellular polysaccharides production is reduced upon treatment with OALC (Fig 7), a lower availability in Psl and Pel is reasonably considered. Although we didn’t quantify Psl production, the hypothesis that OALC targets Psl biosynthesis and/or accumulation in biofilm matrix could explain the disruption in biofilm structures observed in one day-old biofilms treated with OALC (Fig 5B). Indeed, Psl functions as a scaffold, holding cells together in the biofilm matrix [90] and the *psl* operon is also essential for biofilm formation in PAO1 strains, as Psl is involved in both early- and late-stages of biofilm development as well as in the maintenance of biofilm structure so that preformed biofilm can be structurally affected in absence of continuous Psl supply [90, 92]. In contrast, one can assume that the sole inhibition of Pel polysaccharide production could not explain the negative impact in biofilm formation by PAO1 as Δ*pelD* mutant have been shown to not exhibit significant reduction in biofilm formation compared to PAO1 wild type [93]. As a consequence of polysaccharide defects induced by OALC (Fig 7), biofilm biomass is reduced (Fig 4) and planktonic lifestyle is promoted (Fig 4B) and further experiments are now required to determine if OALC promotes biofilm dispersal, particularly production of c-di-GMP, small regulatory RNAs (RsmY and RsmZ) as well as flagellar motility should be investigated as they are intimately implicated in biofilm dispersion [94].

**Fig 10:**
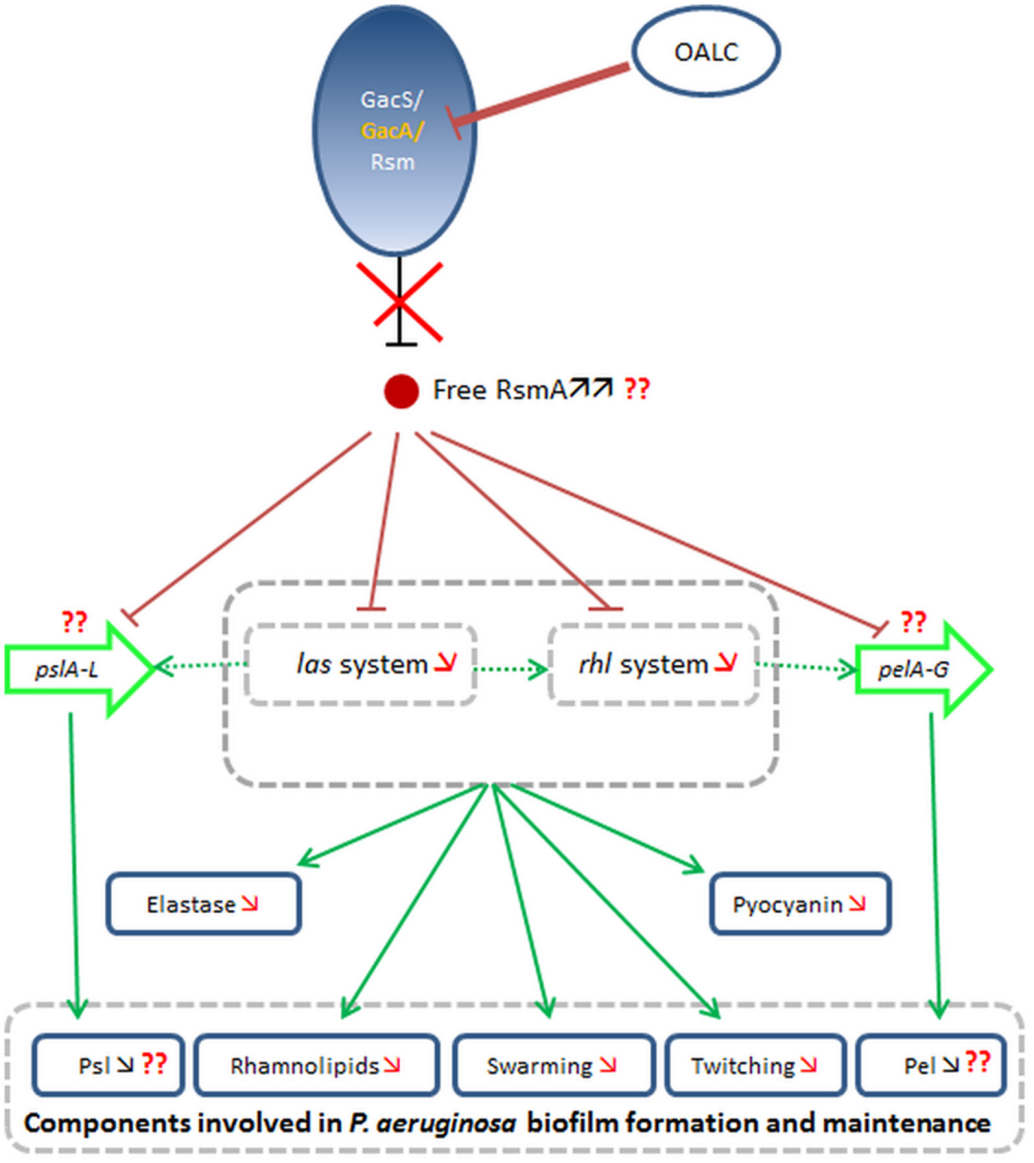
Proposed targets and inhibition cascade occurring in *P*. *aeruginosa* in presence of OALC. In *P*. *aeruginosa*, OALC inhibits *gacA* gene expression (key component of *GacS/GacA/Rsm* signal transduction pathway) leading to an increased level of free RsmA [95]. The latter negatively regulates *las* and *rhl* systems leading to inhibition of rhamnolipids, pyocyanin and elastase production [9] as well as components involved in biofilm formation and maintenance, including motilities (swarming and twitching) and Psl/Pel productions [107] independently of QS systems. Exopolysaccharides production is also reduced directly by RsmA [99].

The negative effect of OALC on *las* and *rhl* systems was correlated with a significant reduction in the corresponding concentrations of inducer signals, 3-oxo-C12-HSL and C4-HSL, respectively (Fig 2). However, the reduction of native AHLs is not compensated by exogenous AHLs supply (S6 Fig), indicating that OALC target is beyond AHLs synthesis in the QS pathways. Therefore, we hypothesized that the starting point for the disruption of QS (inhibition of *las* and *rhl* systems) and biofilm formation (mainly the reduction in polysaccharides and rhamnolipids production as well as bacterial motility) could be associated with the inhibition of *gacA* expression by OALC (see Fig 10 for the proposed inhibition cascade). In this model, the two-component regulatory system *GacS/GacA* that normally inactivate the RNA binding protein, RsmA [95] are presumably impaired at *gacA* gene level. Consequently, an increase of free RsmA should be observed which lead to the repression of *lasI* and *rhlI* gene expression and therefore, the inhibition of bacterial behaviors (including motilities and biofilm formation) and extracellular virulence factors controlled by the *las* and *rhl* systems [96, 97]. Likewise, regulations of Pel and Psl production are also presumably disrupted as high levels of free RsmA repress *psl* and *pel* expressions and therefore disrupt biofilm formation [98, 99].

The *in vitro* ability of OALC to “weaken” the biofilm matrix as a physical barrier protecting encapsulated bacteria cells against antibiotics is probably at the origin of the observed OALC-tobramycin synergistic activity (Figs 8 and 9). As a result, the antibiotics may better penetrate into the biofilm layers, acting efficiently on bacterial cells more than it could be in structured mature biofilm where antibiotics fail to reach entire bacterial population within the matrix. Our data indicate that this OALC-tobramycin synergistic activity could depend on the observed reduction in polysaccharides production (see Fig 7). Consistently, previous studies have shown that PAO1 biofilm polysaccharides (Pel and Psl, particularly) confer protection against aminoglycoside antibiotics [23] and fast-acting antibiotic defense by affecting the interaction between antibiotics and bacterial cell wall [62, 100]. Thus, synergistic effect of OALC is not linked to an increase of bactericidal activity but rather in an increase of availability of tobramycin inside biofilm matrix. Moreover, several studies corroborate that degraded or at least weakened-biofilm by anti-biofilm compounds through anti-QS or other mechanisms enhance antibiotic action against bacterial biofilms although no mechanism has been clearly proposed by authors [101, 102].

Anti-virulence propriety of OALC was further evidenced by using *C*. *elegans*, one of the simplest invertebrate models for studying the *P*. *aeruginosa*-host interactions [103]. Previous studies demonstrated that *P*. *aeruginosa* PAO1 kills *C*. *elegans* but the QS-deficient *lasR* and *lasI rhlI* double mutant strains which fail to produce fully AHLs presumably like PAO1 in presence of OALC (Fig 2), appear strongly attenuated in this virulence model [50, 51]. Herein, pre-incubation of PAO1 with OALC considerably reduced the expression of bacterial pathogenic traits, although the treatment was less efficient compared to the reference compound 4-NPO (S7 Fig).

In this study, although the concentration of 200μM is higher than azithromycin (2.67 μM) and is hardly feasible for clinical treatment [69], OALC appears as a potent chemical backbone candidate for further structure-activity studies. OALC is composed of two moieties; an oleanane-type triterpene bearing a *p*-coumaroyl group. Bodini et al. [104] have demonstrated that *p*-coumarate exhibits a QSI effect in *Chromobacterium violaceum*, *Agrobacterium tumefaciens* and *P*. *putida*. Moreover, other studies indicate that oleanolic acid inhibits the *in vitro* biofilm formation by *S*. *aureus* and *P*. *aeruginosa* [105, 106]. Accordingly, the dual effect of OALC on both QS regulation and biofilm formation could be linked to the coumaroyl and oleanane groups, respectively. Thus, detailed quantitative structure-activity relationship analysis of the various radicals and cycles as well as the substitutions of the ester link might shed light on the chemical functions required for the anti-QS and anti-biofilm activities of OALC and might lead to more active and stable compounds, with reduced toxicity; an important criteria for drug development.

## Supporting Information

S1 Text Chromatographic fractionation of *D*. *trichocarpa* bark extract and isolation of OALC. Following first fractionation of *n*-hexane extracts of *D*. *trichocarpa* barks (residue of 40 g; see experimental procedures for protocol details), nine fractions were collected from the column chromatography. All of them (except fraction 2) were found to inhibit QS-related (*lasB* and *rhlA*) genes expression and/or biofilm formation without affecting bacterial growth (S1A Fig). For further exploration, active fraction products were selected on the basis of the following criteria *(i)* bacterial growth was not affected; *(ii)* anti-QS and anti-biofilm formation activities are noticeable; and *(iii)* amounts of collected residues allowed further fractionation and chemical characterization of the active compound(s). Accordingly, fraction 4 was selected for further fractionation by prep-HLPC to yield the active subfraction F4-5 and F4-7 (S1B Fig). However, only purified compound from subfraction F4-7 (30 mg) was further characterized due to limited quantity of purified compound from subfraction F4-5 (3 mg).
(DOCX)

S2 Text Structural elucidation of the active compound as oleanolic aldehyde coumarate. The active compound was isolated as a white powder and was visualized on TLC plate as a pinkish-purple dark spot following spraying with a vanillin-H2SO4 reagent, suggesting the occurrence of a terpenoid skeleton [108]. The UV spectrum profile (λ_max_: 203, 226, 308.8 nm) suggested the presence of a 4-oxygenated cinnamic acid derivative [109, 110] (S2A Fig). The ESI-MS (*rel*. *int*. *%*) analysis of active compound revealed a [M−H]^−^ pseudo-molecular peak ion at *m*/*z* 585.52 (100), five peaks at *m/z* 293.06 (10), 621.14 (72), 623.15 (32), 1207.25 (18) and at *m*/*z* 1170.98 (30) (S2B Fig). ^1^H-NMR spectrum showed two obvious groups of signals, one group corresponding to a terpenoid moiety and the other group to a cinnamic acid moeity (S3 Fig). One proton showed singlet at δ 9.40 indicating H-28 in carbonyl carbon; the typical signals for the olefinic double bond H-12 of the structure were obvious from one broad singlet (or t-like) at δ 5.34 (1H) and one proton doublet of doublets at δ 4.61 (*J* = 8.80, 7.60 Hz), suggesting the presence of H-3. Two doublets (1H each) at δ 6.30 (*J* = 16.0 Hz) and δ 7.61 (*J* = 16.0 Hz) are evidence of the presence of trans-coupled protons H-8' and H-7', respectively. Two doublets (2H each) at δ 6.82 (*J* = 8.4 Hz) and δ 7.43 (*J* = 8.4 Hz) are assignable to aromatic protons H-3', 5' and H-2', 6', respectively [111, 112]. The ^13^C NMR spectrum of the active compound also exhibited characteristic signals for one ester carbonyl group at δ 167.4 (S2 Table), four olefinic carbons at δ 123.4 (C-12), δ 143.2 (C-13), 144.3 (C-7’) and δ 116.4 (C-8’), four aromatic signals at δ 116.1 (C-3’, 5’), δ 127.4 (C-1’), δ 130.1 (C-2’, 6’), and δ 158.0 (C-4’), and one hydroxylated carbons at δ 81.0 (C-3). At this stage, by comparison of their physical and spectroscopic data (^1^H-NMR, ^13^C-NMR) with those reported in the literature, the above-described characteristics of active compound present some similarity to cinnamoyl triterpenes, such as the *p*-coumaroyloleanolic acid isolated from *Hippophae rhamnoides* branch bark and *Bauhinia purpurea* bark [78, 113] and, more recently, the (3β)-(4-hydroxy-*E*-cinnamoyl) olean-5,12-dien-28-ol isolated from *Crotalaria incana* leaves [114]. Finally, unambiguous assignment of the active compound was achieved through 2D NMR [^1^H-^1^H correlation spectroscopy (COSY), heteronuclear single quantum coherence (HSQC), heteronuclear multiple bond correlation (HMBC) and nuclear overhauser effect spectroscopy (NOESY)] experiments (S3 Fig). Based on these spectroscopic data, the active compound was identified as a 3β-hydroxyolean-12-en-28-al 3-*p*-coumarate or oleanolic aldehyde coumarate (OALC, C_39_H_54_O_4_) (Fig 1).
(DOCX)

S1 Table *P*. *aeruginosa* strains and plasmids used in this study. (DOCX)

S2 Table ^1^H- and ^13^C-NMR spectral data of 3β-hydroxyolean-12-en-28-al 3-*p*-coumarate (400/400 MHz, CDCl_3_, δ (ppm) (*J* = Hz)). (DOCX)

S3 Table Construction of plamids pLP170_*gacA* and pLP170_*vfr*. (DOCX)

S1 Fig Chromatographic scheme of *D*. *trichocarpa* bark fractionation steps used to isolate the major QS and biofilm inhibitory compound. (A) Silica gel column chromatography eluted with hexane/EtOAc gradient mixture (10:0–0:10) and then EtOAc/MeOH (10:0–0:10). Bioactivity was monitored using QS-related (*lasB* and *rhlA*) genes expression and biofilm formation; significant inhibition activity was scored “+”. Active fraction 4 was eluted from the column chromatography with the solvent mixture hexane/EtOAc (7:3). (B) High performance liquid chromatogram profile of active fraction F4 monitored at 300 nm. HPLC conditions: injection, 20 μg; column, RP Atlantis dC18 5μm (4.6 by 250 mm); H_2_O-acetonitrile gradient (10:90 in 5 min, 10:90 to 0:100 in 12 min, 0:100 in 3 min, 0:100 to 10:90 in 2 min, 10:90 in 8 min); 1 mL min^-1^. Activity was monitored using QS-related gene expression and biofilm formation. Significant inhibition activity was shown for subfraction F4-5 and F4-7.
(TIF)

S2 Fig UV and mass spectra of OALC. (A) UV spectrum of OALC. (B) Electrospray ionization mass spectrum of OALC. Mass spectrum was acquired by direct injection into an electrospray ionization source operated in the negative mode on Finnigan LCQ DUO mass spectrometer. Scans were averaged over 1 min with following conditions: solvent acetonitrile; concentration loaded 10 μg mL^-1^; negative ionization mode; nebulizer tip set at 250°C and 4.52 kV; cone voltage set at 5 kV; sheath gas (nitrogen) flow rate at 28 arbitrary units; collision energy at -70 eV; MS data were acquired in the *m/z* range from 50 to 2000.
(TIF)

S3 Fig NMR spectrum of OALC. (A) ^1^H NMR spectrum of OALC in CDCl_3_ (400 MHz). (B) ^13^C NMR spectrum of OALC in CDCl_3_ (400 MHz). (C) HSQC NMR spectrum of OALC in CDCl_3_ (400 MHz). (D) HMBC NMR spectrum of OALC in CDCl_3_ (400 MHz). (E) COSY NMR spectrum of OALC in CDCl_3_ (400 MHz). (F) NOESY NMR spectrum of OALC in CDCl_3_ (400 MHz).
(PDF)

S4 Fig Effect of OALC at different concentrations (50 to 200 μM) on the expression of PAO1 QS-regulated genes *lasB* and *rhlA* and QS independent gene *aceA* after 18 hours of incubation. (A) Effect of OALC on QS-regulated *lasB* gene expression. (B) Effect of OALC on QS-regulated *rhlA* gene expression. (C) Effect of OALC on QS independent *aceA* gene expression. The cell density of the bacteria was assessed at 600 nm (clear bar) and gene expression was measured as the β-galactosidase activity of the *lacZ* gene fusions and expressed in Miller units (grey bar). Naringenin (Nar, 4 mM) is used as a quorum sensing inhibitor control and naringin (Nin, 4 mM) as a negative control. Error bars represent the standard errors of the means and all experiments were performed in quintuplicate with three independent assays and asterisks indicate samples that are significantly different from the DMSO (Student’s *t*-tests; *P* ≤ 0.01).
(TIF)

S5 Fig Growth kinetics of *P*. *aeruginosa* PAO1 in the presence of DMSO 1% or 200 μM OALC. PAO1 cell viability was assessed after 8h and 18 h by C.F.U. measurement. The statistical significance of each test (n = 5) was evaluated by Student’s t test (i.e. each test was compared with the DMSO condition), and a *P* value of < 0.01 was considered significant.
(TIF)

S6 Fig Effectof OALC on the production of (A) elastase and (B) pyocyanin after exogenous supply of homoserine lactones to wild type strain PAO1. Effect of OALC on the production of (C) elastase and (D) pyocyanin after exogenous supply of the appropriate AHLs to mutant strains, respectively, Δ*lasI* (ΔPA1432, mutant ID 11174) and Δ*rhlI* (ΔPA3476, mutant ID 32454). Productions of pyocyanin and elastase were quantified as in Fig 2. In each case, bacteria were incubated with DMSO, naringenin (Nar), naringin (Nin), OALC, C4-HSL or 3-oxo-C12-HSL). Bacteria were also induced with the appropriate AHL and simultaneously treated with naringenin (+ Nar) or (+Nin) or OALC (+ OALC). C4-HSL and 3-oxo-C12-HSL were added at 10 μM final concentration. DMSO-treated cultures were used as controls, the statistical significance of each test (n = 5) was evaluated by conducting one-way ANOVA with Tukey’s multiple comparison tests, and a *P* value of ≤ 0.01 was considered significant (asterisks indicate samples that are significantly different from the DMSO). For mutant Δ*lasI* and Δ*rhlI*, the letters above the histograms indicate that the data are statistically different from each other (*P* ≤ 0.01).
(TIF)

S7 Fig Mortality of *C*. *elegans* nematodes living on a lawn of PAO1 and reduced virulent strains treated with DMSO 1%, naringenin (4 mM), naringin (4 mM) or OALC (200 μM). (A) Mortality of *C*. *elegans* nematodes living on a lawn of *P*. *aeruginosa* PAO1 treated with DMSO 1%, naringenin (4 mM), naringin (4 mM) or OALC (200 μM). (B) Mortality of *C*. *elegans* nematodes living on a lawn of ΔPA1430 (*ΔlasR*). (C) Mortality of *C*. *elegans* nematodes living on a lawn of ΔPA3477 (*ΔrhlR*). (D) Toxicity effect of OALC at different concentration (100 μM, 200 μM or 300 μM) and naringenin at 4mM (See experimental procedures for details). Bars show an average of five experiments, and errors bars indicate the standard deviation between experiments. *, data that are statistically different (*p* ≤ 0.01).
(TIF)
