# Supplementary material for: Pseudomonas aeruginosa Biofilm Formation and Persistence, along with the Production of Quorum Sensing-Dependent Virulence Factors, Are Disrupted by a Triterpenoid Coumarate Ester Isolated from Dalbergia trichocarpa, a Tropical Legume
Source: PLoS One. 2015 Jul 17;10(7):e0132791. doi: 10.1371/journal.pone.0132791 (PMC4505864; doi:10.1371/journal.pone.0132791)
Supplement: S1 Text — Following first fractionation of n-hexane extracts of D. trichocarpa barks (residue of 40 g; see experimental procedures for protocol details), nine fractions were collected from the column chromatography. All of them (except fraction 2) were found to inhibit QS-related (lasB and rhlA) genes expression and/or biofilm formation without affecting bacterial growth (S1A Fig). For further exploration, active fraction products were selected on the basis of the following criteria (i) bacterial growth was not affected; (ii) anti-QS and anti-biofilm formation activities are noticeable; and (iii) amounts of collected residues allowed further fractionation and chemical characterization of the active compound(s). Accordingly, fraction 4 was selected for further fractionation by prep-HLPC to yield the active subfraction F4-5 and F4-7 (S1B Fig). However, only purified compound from subfraction F4-7 (30 mg) was further characterized due to limited quantity of purified compound from subfraction F4-5 (3 mg). (DOCX) [file pone.0132791.s001.docx]

**S1 Text. Chromatographic fractionation of *D. trichocarpa* bark extract and isolation of OALC**

Following first fractionation of *n*-hexane extracts of *D. trichocarpa* barks (residue of 40 g; see experimental procedures for protocol details), nine fractions were collected from the column chromatography. All of them (except fraction 2) were found to inhibit QS-related (*lasB* and *rhlA*) genes expression and/or biofilm formation without affecting bacterial growth (S1A Fig). For further exploration, active fraction products were selected on the basis of the following criteria *(i)* bacterial growth was not affected; *(ii)* anti-QS and anti-biofilm formation activities are noticeable; and *(iii)* amounts of collected residues allowed further fractionation and chemical characterization of the active compound(s). Accordingly, fraction 4 was selected for further fractionation by prep-HLPC to yield the active subfraction F4-5 and F4-7 (S1B Fig). However, only purified compound from subfraction F4-7 (30 mg) was further characterized due to limited quantity of purified compound from subfraction F4-5 (3 mg).
