# Supplementary material for: Pseudomonas aeruginosa Biofilm Formation and Persistence, along with the Production of Quorum Sensing-Dependent Virulence Factors, Are Disrupted by a Triterpenoid Coumarate Ester Isolated from Dalbergia trichocarpa, a Tropical Legume
Source: PLoS One. 2015 Jul 17;10(7):e0132791. doi: 10.1371/journal.pone.0132791 (PMC4505864; doi:10.1371/journal.pone.0132791)
Supplement: S2 Text — The active compound was isolated as a white powder and was visualized on TLC plate as a pinkish-purple dark spot following spraying with a vanillin-H2SO4 reagent, suggesting the occurrence of a terpenoid skeleton [108]. The UV spectrum profile (λmax: 203, 226, 308.8 nm) suggested the presence of a 4-oxygenated cinnamic acid derivative [109, 110] (S2A Fig). The ESI-MS (rel. int. %) analysis of active compound revealed a [M−H]− pseudo-molecular peak ion at m/z 585.52 (100), five peaks at m/z 293.06 (10), 621.14 (72), 623.15 (32), 1207.25 (18) and at m/z 1170.98 (30) (S2B Fig). 1H-NMR spectrum showed two obvious groups of signals, one group corresponding to a terpenoid moiety and the other group to a cinnamic acid moeity (S3 Fig). One proton showed singlet at δ 9.40 indicating H-28 in carbonyl carbon; the typical signals for the olefinic double bond H-12 of the structure were obvious from one broad singlet (or t-like) at δ 5.34 (1H) and one proton doublet of doublets at δ 4.61 (J = 8.80, 7.60 Hz), suggesting the presence of H-3. Two doublets (1H each) at δ 6.30 (J = 16.0 Hz) and δ 7.61 (J = 16.0 Hz) are evidence of the presence of trans-coupled protons H-8' and H-7', respectively. Two doublets (2H each) at δ 6.82 (J = 8.4 Hz) and δ 7.43 (J = 8.4 Hz) are assignable to aromatic protons H-3', 5' and H-2', 6', respectively [111, 112]. The 13C NMR spectrum of the active compound also exhibited characteristic signals for one ester carbonyl group at δ 167.4 (S2 Table), four olefinic carbons at δ 123.4 (C-12), δ 143.2 (C-13), 144.3 (C-7’) and δ 116.4 (C-8’), four aromatic signals at δ 116.1 (C-3’, 5’), δ 127.4 (C-1’), δ 130.1 (C-2’, 6’), and δ 158.0 (C-4’), and one hydroxylated carbons at δ 81.0 (C-3). At this stage, by comparison of their physical and spectroscopic data (1H-NMR, 13C-NMR) with those reported in the literature, the above-described characteristics of active compound present some similarity to cinnamoyl triterpenes, such as the p-coumaroyloleanolic acid isolated [file pone.0132791.s002.docx]

**S2 Text. Structural elucidation of the active compound as oleanolic aldehyde coumarate**

The active compound was isolated as a white powder and was visualized on TLC plate as a pinkish-purple dark spot following spraying with a vanillin-H2SO4 reagent, suggesting the occurrence of a terpenoid skeleton [108]. The UV spectrum profile (λ_max_: 203, 226, 308.8 nm) suggested the presence of a 4-oxygenated cinnamic acid derivative [109, 110] (S2A Fig). The ESI-MS (*rel. int. %*) analysis of active compound revealed a [M−H]^−^ pseudo-molecular peak ion at *m*/*z* 585.52 (100), five peaks at *m/z* 293.06 (10), 621.14 (72), 623.15 (32), 1207.25 (18) and at *m*/*z* 1170.98 (30) (S2B Fig). ^1^H-NMR spectrum showed two obvious groups of signals, one group corresponding to a terpenoid moiety and the other group to a cinnamic acid moeity (S3 Fig). One proton showed singlet at δ 9.40 indicating H-28 in carbonyl carbon; the typical signals for the olefinic double bond H-12 of the structure were obvious from one broad singlet (or t-like) at δ 5.34 (1H) and one proton doublet of doublets at δ 4.61 (*J* = 8.80, 7.60 Hz), suggesting the presence of H-3. Two doublets (1H each) at δ 6.30 (*J* = 16.0 Hz) and δ 7.61 (*J* = 16.0 Hz) are evidence of the presence of trans-coupled protons H-8' and H-7', respectively. Two doublets (2H each) at δ 6.82 (*J* = 8.4 Hz) and δ 7.43 (*J* = 8.4 Hz) are assignable to aromatic protons H-3', 5' and H-2', 6', respectively [111, 112]. The ^13^C NMR spectrum of the active compound also exhibited characteristic signals for one ester carbonyl group at δ 167.4 (S1 Table ), four olefinic carbons at δ 123.4 (C-12), δ 143.2 (C-13), 144.3 (C-7’) and δ 116.4 (C-8’), four aromatic signals at δ 116.1 (C-3’, 5’), δ 127.4 (C-1’), δ 130.1 (C-2’, 6’), and δ 158.0 (C-4’), and one hydroxylated carbons at δ 81.0 (C-3). At this stage, by comparison of their physical and spectroscopic data (^1^H-NMR, ^13^C-NMR) with those reported in the literature, the above-described characteristics of active compound present some similarity to cinnamoyl triterpenes, such as the *p*-coumaroyloleanolic acid isolated from *Hippophae rhamnoides* branch bark and *Bauhinia purpurea* bark [78, 113] and, more recently, the (3β)-(4-hydroxy-*E*-cinnamoyl) olean-5,12-dien-28-ol isolated from *Crotalaria incana* leaves [114]. Finally, unambiguous assignment of the active compound was achieved through 2D NMR [^1^H-^1^H correlation spectroscopy (COSY), heteronuclear single quantum coherence (HSQC), heteronuclear multiple bond correlation (HMBC) and nuclear overhauser effect spectroscopy (NOESY)] experiments (S3 Fig). Based on these spectroscopic data, the active compound was identified as a 3β-hydroxyolean-12-en-28-al 3-*p*-coumarate or oleanolic aldehyde coumarate (OALC, C_39_H_54_O_4_) (Fig 1).
