# Supplementary material for: Pseudomonas aeruginosa Biofilm Formation and Persistence, along with the Production of Quorum Sensing-Dependent Virulence Factors, Are Disrupted by a Triterpenoid Coumarate Ester Isolated from Dalbergia trichocarpa, a Tropical Legume
Source: PLoS One. 2015 Jul 17;10(7):e0132791. doi: 10.1371/journal.pone.0132791 (PMC4505864; doi:10.1371/journal.pone.0132791)
Supplement: S1 Table — (DOCX) [file pone.0132791.s003.docx]

**S1 Table.** *Pseudomonas aeruginosa* strains and plasmids used in this study.

| Strains or plasmids | Relevant characteristics | References |
| --- | --- | --- |
| Strains |  |  |
| *P. aeruginosa* PAO1 | Wild-type (strain PAO0001; http://www.pseudomonas.med.ecu.edu/) |  |
| *P. aeruginosa* ∆PA1430 | *P. aeruginosa* transposon mutant ID17281; *lasR::*IS*lacZ/*hah;Tet^R^ | [32] |
| *P. aeruginosa* ∆PA1432 | *P. aeruginosa* transposon mutant ID11174; *lasI::*IS*lacZ/*hah;Tet^R^ | [32] |
| *P. aeruginosa* ∆PA3476 | *P. aeruginosa* transposon mutant ID32454; *rhlI::*IS*phoA/*hah;Tet^R^ | [32] |
| *P. aeruginosa* ∆PA3477 | *P. aeruginosa* transposon mutant ID3452; *rhlR::*IS*lacZ/*hah;Tet^R^ | [32] |
| Plasmids |  |  |
| pLP170 | Broad-host-range *lacZ* transcriptional fusion vector containing an RNase III splice sequence positioned between the multiple cloning site and *lacZ*; Cb^r^ | [115] |
| pPCS1001 | pLP170-derivative containing P*_lasR_-lacZ* transcriptional fusion | [115] |
| pLPR1 | pLP170-derivative containing P*_rhlI_-lacZ* transcriptional fusion | [116] |
| pPCS1002 | pLP170-derivative containing P*_rhlR_-lacZ* transcriptional fusion | [115] |
| pLP170_*gacA* | pLP170- derivative containing P*_gacA_-lacZ* transcriptional fusion | This study* |
| pLP170_*vfr* | pLP170- derivative containing P*_vfr_-lacZ* transcriptional fusion | This study* |
| pQF50 | Broad-host-range promoter-less *lacZ* transcriptional fusion vector; Cb^r^ | [39] |
| pβ01 | pQF50-derivative containing P*_lasB_-lacZ* transcriptional fusion | [39] |
| pβ02 | pQF50-derivative containing P*_rhlA_-lacZ* transcriptional fusion | [39] |
| pβ03 | pQF50-derivative containing P*_lasI_-lacZ* transcriptional fusion | [39] |
| pTB4124 | pQF50-derivative containing P*_aceA_-lacZ* transcriptional fusion | [36] |

* Primer sequences used to construct *gacA* and *vfr* plasmids are reported as supporting information (S3 Table)
