# Supplementary material for: Pseudomonas aeruginosa Biofilm Formation and Persistence, along with the Production of Quorum Sensing-Dependent Virulence Factors, Are Disrupted by a Triterpenoid Coumarate Ester Isolated from Dalbergia trichocarpa, a Tropical Legume
Source: PLoS One. 2015 Jul 17;10(7):e0132791. doi: 10.1371/journal.pone.0132791 (PMC4505864; doi:10.1371/journal.pone.0132791)
Supplement: S2 Table — (DOCX) [file pone.0132791.s004.docx]

**S2 Table. ^1^H- and ^13^C-NMR spectral data of 3β-hydroxyolean-12-en-28-al 3-*p*-coumarate (400/400 MHz, CDCl_3_, δ (ppm) (*J*=Hz)).**

| Position | δ_C_ | δ_H_ | | Position | δ_C_ | δ_H_ | |
| --- | --- | --- | --- | --- | --- | --- | --- |
|  |  | Ha | Hb |  |  | Ha | Hb |
| 1 | 38.4 | 1.61 m | 1.05 m | 21 | 33.3 | 1.27 m | 1.27 m |
| 2 | 23.9 | 1.66 m | 1.66 m | 22 | 28.0 | 1.43 m | 1.18 m |
| 3 | 81.0 | 4.61 (bt, ^3^*J*2-3 = 8.0) | / | 23 | 28.3 | 0.87 (s, 3H) | / |
| 4 | 38.2 | / | / | 24 | 17.1 | 0.90 (s, 3H) | / |
| 5 | 55.5 | 0.84 m | / | 25 | 15.6 | 0.93 (s, 3H) | / |
| 6 | 18.4 | 1.51 m | 1.34 m | 26 | 17.2 | 0.72 (s, 3H) | / |
| 7 | 32.9 | 1.4 m | 1.4 m | 27 | 25.7 | 1.12 (s, 3H) | / |
| 8 | 39.8 | / | / | 28 | 207.9 | 9.38 (s, 1H) | / |
| 9 | 47.7 | 1.53 m | / | 29 | 33.4 | 0.89 (s, 3H) | / |
| 10 | 37.1 | / | / | 30 | 23.6 | 0.89 (s, 3H) | / |
| 11 | 23.6 | 1.86 m | 1.86 m | 1’ | 127.4 | / | / |
| 12 | 123.4 | 5.32 (t, ^3^*J*11-12 = 3.4) | / | 2’ | 130.1 | 7.41 (d, ^3^*J*2'-3' = 8.5) | / |
| 13 | 143.2 | / | / | 3’ | 116.1 | 6.81 (d, ^3^*J*2'-3' = 8.5) | / |
| 14 | 41.9 | / | / | 4’ | 158.0 | / | / |
| 15 | 27.0 | 1.62 m | 1.05 m | 5’ | 116.1 | 6.81 (d, ^3^*J*5'-6' = 8.5) | / |
| 16 | 22.3 | 1.96 m | 1.52 m | 6’ | 130.1 | 7.41 (d, ^3^*J*5'-6' = 8.5) | / |
| 17 | 49.3 | / | / | 7’ | 144.3 | 7.58 (d, ^3^*J*7'-8' = 15.9) | / |
| 18 | 40.6 | 2.60 (dd, ^3^*J*18-19a = 13.3, ^3^*J*18-19b = 3.9) | / | 8’ | 116.4 | 6.27 (d, ^3^*J*7'-8' = 15.9) | / |
| 19 | 45.8 | 1.66 m | 1.18 m | 9’ | 167.5 | / | / |
| 20 | 30.9 | / | / |  |  |  |  |
