# Supplementary material for: Pseudomonas aeruginosa Biofilm Formation and Persistence, along with the Production of Quorum Sensing-Dependent Virulence Factors, Are Disrupted by a Triterpenoid Coumarate Ester Isolated from Dalbergia trichocarpa, a Tropical Legume
Source: PLoS One. 2015 Jul 17;10(7):e0132791. doi: 10.1371/journal.pone.0132791 (PMC4505864; doi:10.1371/journal.pone.0132791)
Supplement: S3 Table — (DOCX) [file pone.0132791.s005.docx]

**S3 Table.** Construction of plamids pLP170_*gacA* and pLP170_*vfr*

| The promoter region of gacA was amplified from the P. aeruginosa PAO1 genome by double PCR with oligonucleotides 5′-GGCTGAGGAATACCCTCGTTCG-3′, 5′- AATTGGCTGAGGAATACCCTCGTTCG-3′ and 5′- CAGACAGTCTTCACCGCAGTCG-3′. The PCR product containing cohesive restriction site of *Eco*RI was cloned into pLP170 to construct pLP170_*gacA*. |
| --- |
| The promoter region of vfr was amplified from the P. aeruginosa PAO1 genome by double PCR with oligonucleotides 5′-AGTACGGGATCACAGTCCTGATAGC-3′, 5′-AATTAGTACGGGATCACAGTCCTGATAGC-3′, and 5′-GGAACTTCGCATAGCTGATCTCG-3′. The PCR product containing cohesive restriction site of *Eco*RI was cloned into pLP170 to construct pLP170_*vfr*. |
