# Supplementary figures and images for: Pseudomonas aeruginosa Biofilm Formation and Persistence, along with the Production of Quorum Sensing-Dependent Virulence Factors, Are Disrupted by a Triterpenoid Coumarate Ester Isolated from Dalbergia trichocarpa, a Tropical Legume
Source: PLoS One. 2015 Jul 17;10(7):e0132791. doi: 10.1371/journal.pone.0132791 (PMC4505864; doi:10.1371/journal.pone.0132791)

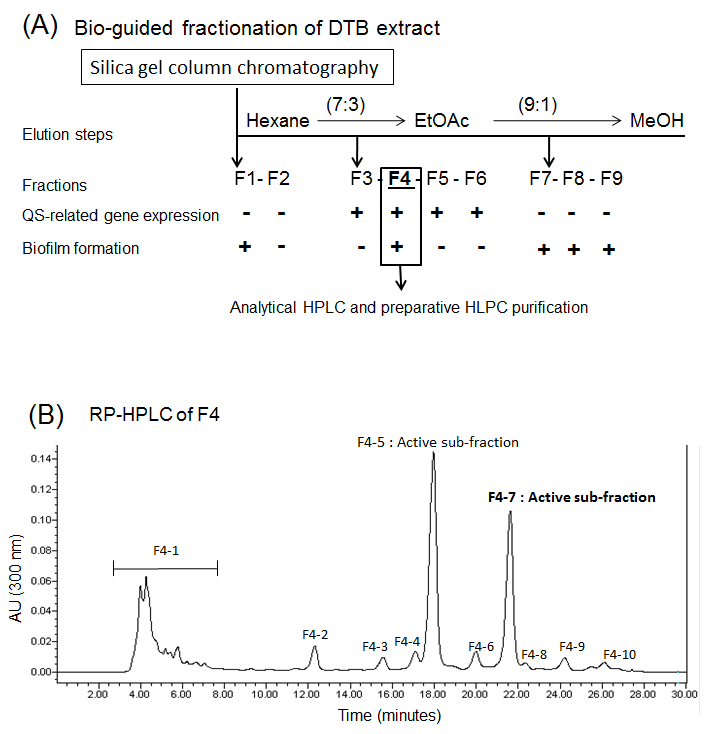

Supplement: S1 Fig — (A) Silica gel column chromatography eluted with hexane/EtOAc gradient mixture (10:0–0:10) and then EtOAc/MeOH (10:0–0:10). Bioactivity was monitored using QS-related (lasB and rhlA) genes expression and biofilm formation; significant inhibition activity was scored “+”. Active fraction 4 was eluted from the column chromatography with the solvent mixture hexane/EtOAc (7:3). (B) High performance liquid chromatogram profile of active fraction F4 monitored at 300 nm. HPLC conditions: injection, 20 μg; column, RP Atlantis dC18 5μm (4.6 by 250 mm); H2O-acetonitrile gradient (10:90 in 5 min, 10:90 to 0:100 in 12 min, 0:100 in 3 min, 0:100 to 10:90 in 2 min, 10:90 in 8 min); 1 mL min-1. Activity was monitored using QS-related gene expression and biofilm formation. Significant inhibition activity was shown for subfraction F4-5 and F4-7. (TIF) [file pone.0132791.s006.tif]

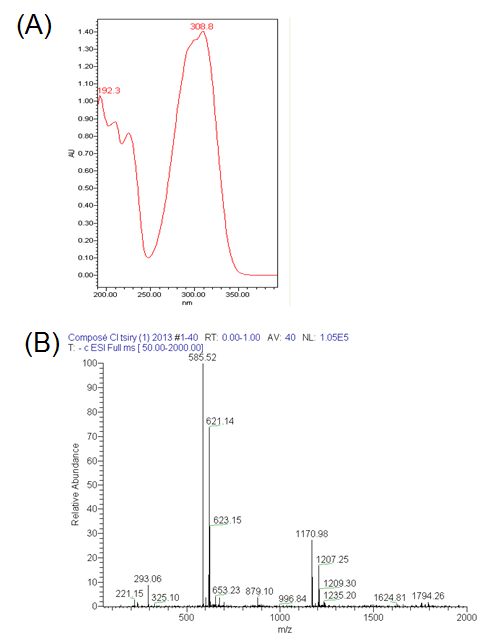

Supplement: S2 Fig — (A) UV spectrum of OALC. (B) Electrospray ionization mass spectrum of OALC. Mass spectrum was acquired by direct injection into an electrospray ionization source operated in the negative mode on Finnigan LCQ DUO mass spectrometer. Scans were averaged over 1 min with following conditions: solvent acetonitrile; concentration loaded 10 μg mL-1; negative ionization mode; nebulizer tip set at 250°C and 4.52 kV; cone voltage set at 5 kV; sheath gas (nitrogen) flow rate at 28 arbitrary units; collision energy at -70 eV; MS data were acquired in the m/z range from 50 to 2000. (TIF) [file pone.0132791.s007.tif]

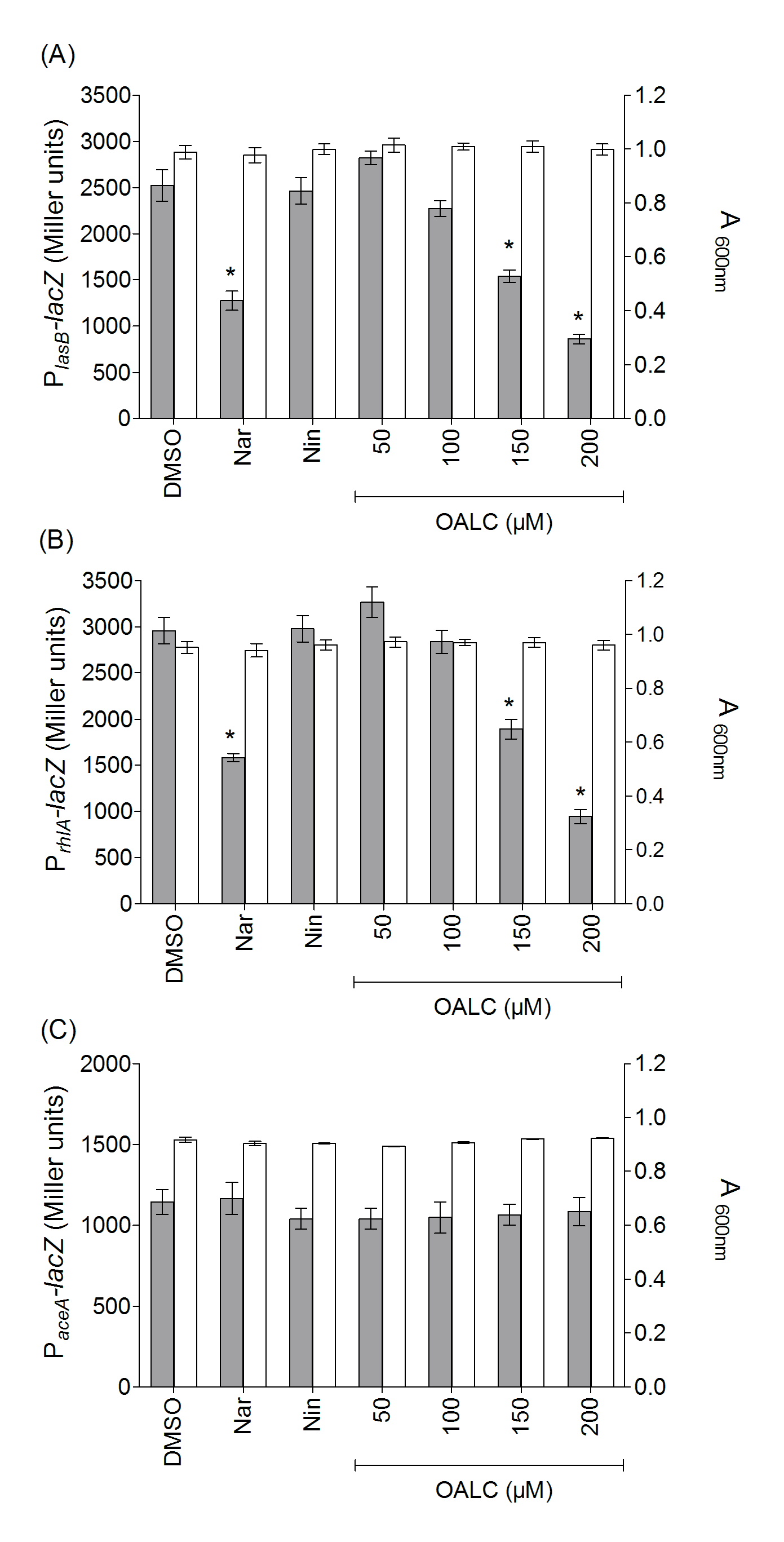

Supplement: S4 Fig — (A) Effect of OALC on QS-regulated lasB gene expression. (B) Effect of OALC on QS-regulated rhlA gene expression. (C) Effect of OALC on QS independent aceA gene expression. The cell density of the bacteria was assessed at 600 nm (clear bar) and gene expression was measured as the β-galactosidase activity of the lacZ gene fusions and expressed in Miller units (grey bar). Naringenin (Nar, 4 mM) is used as a quorum sensing inhibitor control and naringin (Nin, 4 mM) as a negative control. Error bars represent the standard errors of the means and all experiments were performed in quintuplicate with three independent assays and asterisks indicate samples that are significantly different from the DMSO (Student’s t-tests; P ≤ 0.01). (TIF) [file pone.0132791.s009.tif]

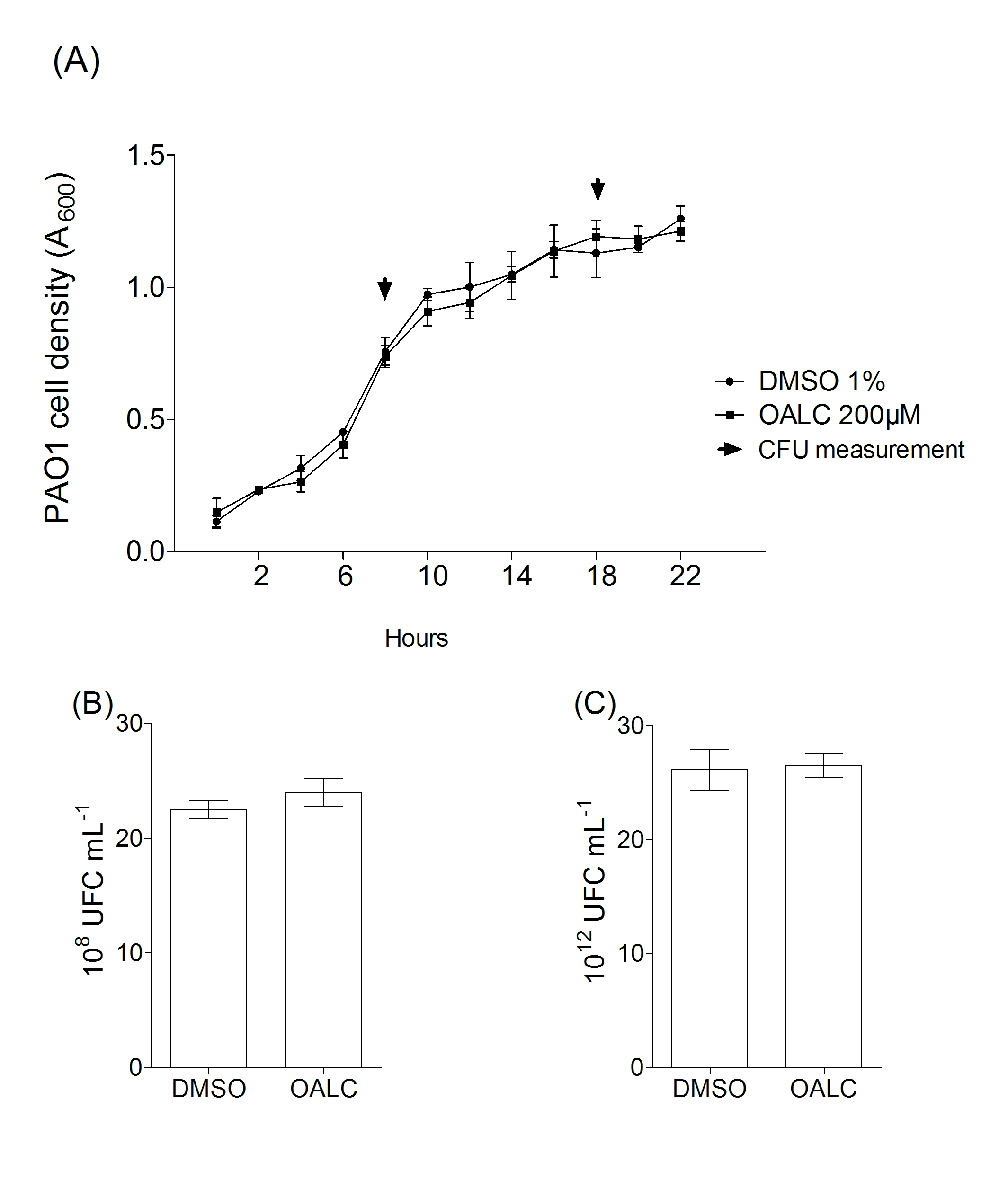

Supplement: S5 Fig — PAO1 cell viability was assessed after 8h and 18 h by C.F.U. measurement. The statistical significance of each test (n = 5) was evaluated by Student’s t test (i.e. each test was compared with the DMSO condition), and a P value of < 0.01 was considered significant. (TIF) [file pone.0132791.s010.tif]

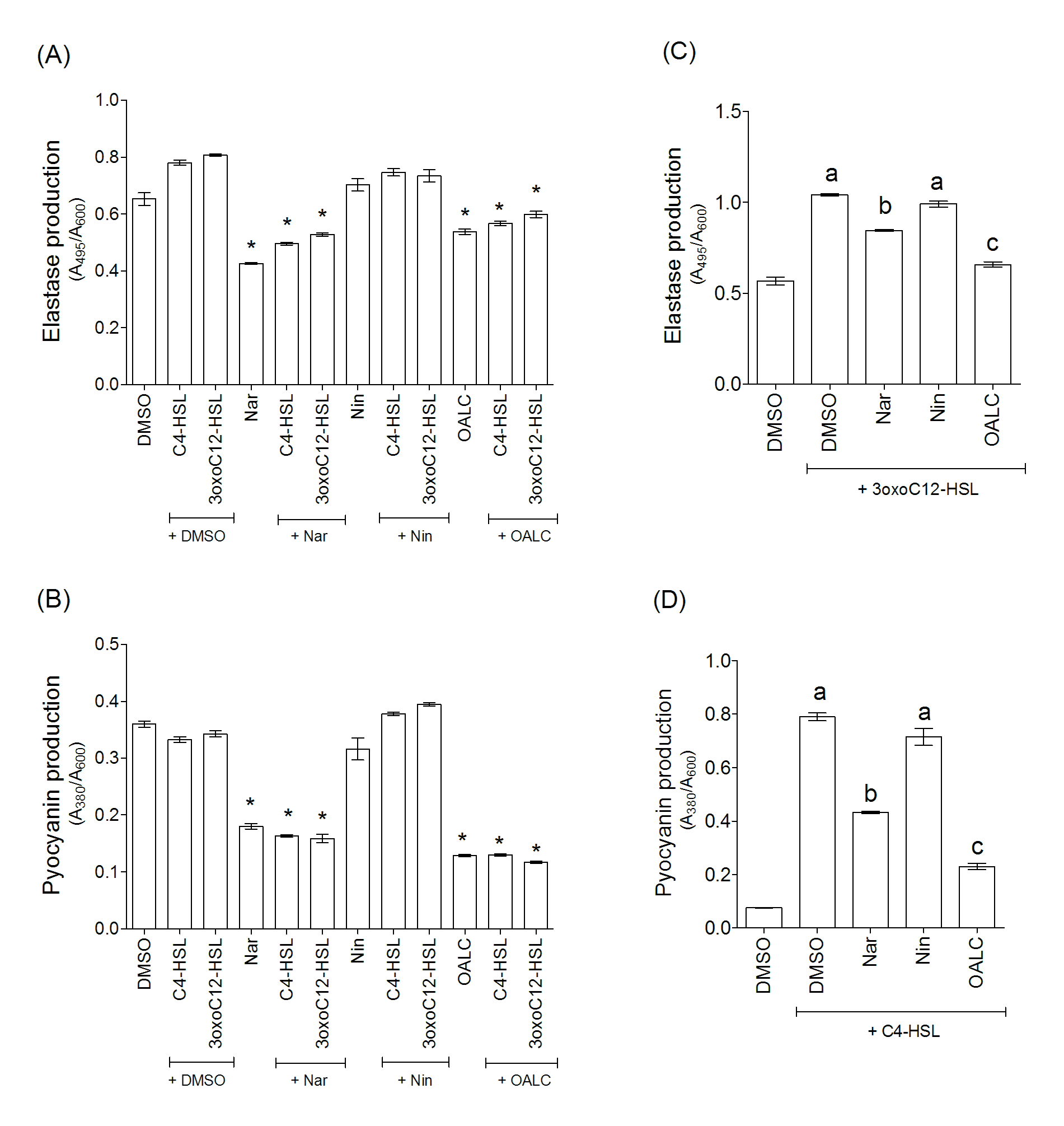

Supplement: S6 Fig — Productions of pyocyanin and elastase were quantified as in Fig 2. In each case, bacteria were incubated with DMSO, naringenin (Nar), naringin (Nin), OALC, C4-HSL or 3-oxo-C12-HSL). Bacteria were also induced with the appropriate AHL and simultaneously treated with naringenin (+ Nar) or (+Nin) or OALC (+ OALC). C4-HSL and 3-oxo-C12-HSL were added at 10 μM final concentration. DMSO-treated cultures were used as controls, the statistical significance of each test (n = 5) was evaluated by conducting one-way ANOVA with Tukey’s multiple comparison tests, and a P value of ≤ 0.01 was considered significant (asterisks indicate samples that are significantly different from the DMSO). For mutant ΔlasI and ΔrhlI, the letters above the histograms indicate that the data are statistically different from each other (P ≤ 0.01). (TIF) [file pone.0132791.s011.tif]

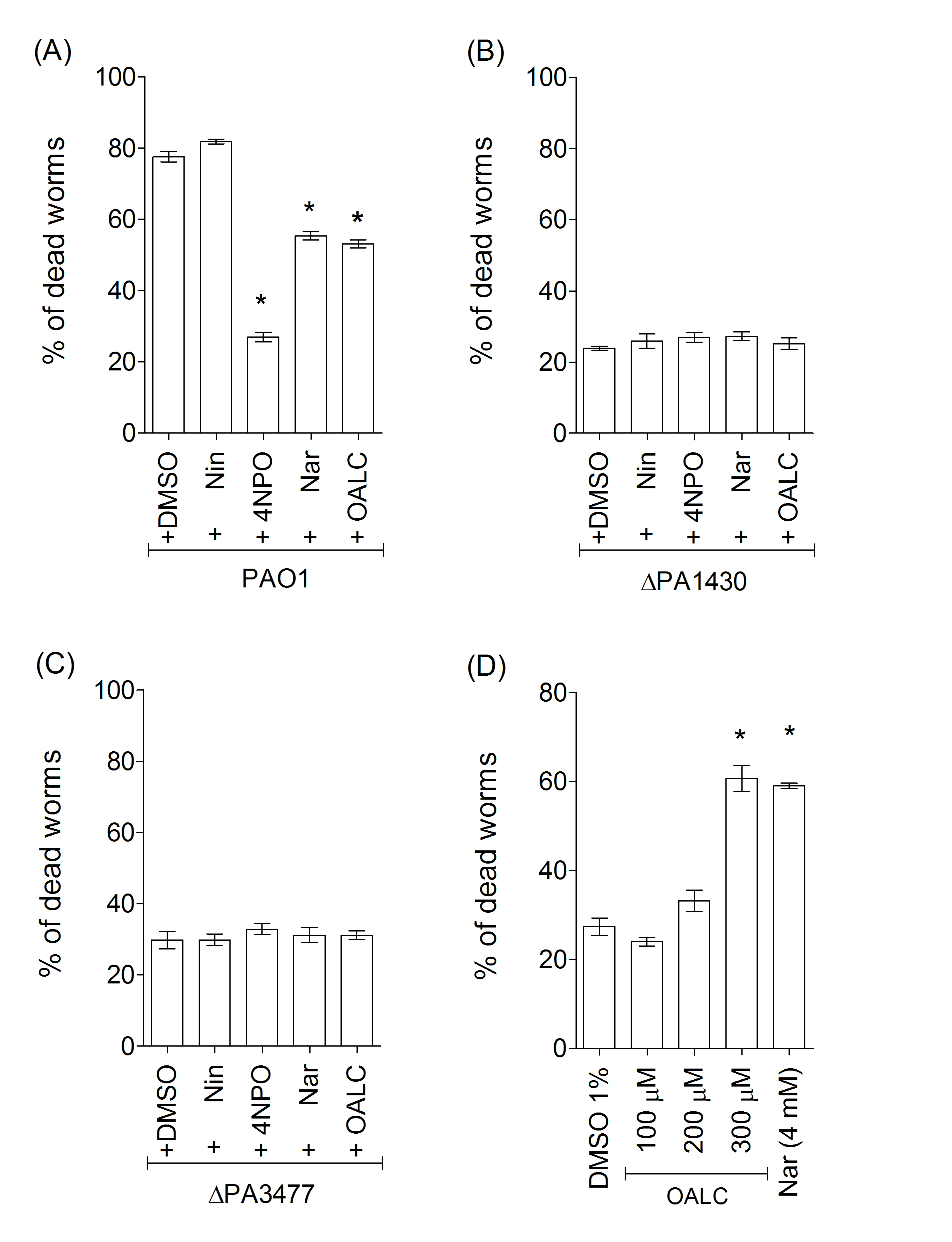

Supplement: S7 Fig — (A) Mortality of C. elegans nematodes living on a lawn of P. aeruginosa PAO1 treated with DMSO 1%, naringenin (4 mM), naringin (4 mM) or OALC (200 μM). (B) Mortality of C. elegans nematodes living on a lawn of ΔPA1430 (ΔlasR). (C) Mortality of C. elegans nematodes living on a lawn of ΔPA3477 (ΔrhlR). (D) Toxicity effect of OALC at different concentration (100 μM, 200 μM or 300 μM) and naringenin at 4mM (See experimental procedures for details). Bars show an average of five experiments, and errors bars indicate the standard deviation between experiments. *, data that are statistically different (p ≤ 0.01). (TIF) [file pone.0132791.s012.tif]
