# Supplementary material for: Pseudomonas aeruginosa Biofilm Formation and Persistence, along with the Production of Quorum Sensing-Dependent Virulence Factors, Are Disrupted by a Triterpenoid Coumarate Ester Isolated from Dalbergia trichocarpa, a Tropical Legume
Source: PLoS One. 2015 Jul 17;10(7):e0132791. doi: 10.1371/journal.pone.0132791 (PMC4505864; doi:10.1371/journal.pone.0132791)
Supplement: S3 Fig — (A) 1H NMR spectrum of OALC in CDCl3 (400 MHz). (B) 13C NMR spectrum of OALC in CDCl3 (400 MHz). (C) HSQC NMR spectrum of OALC in CDCl3 (400 MHz). (D) HMBC NMR spectrum of OALC in CDCl3 (400 MHz). (E) COSY NMR spectrum of OALC in CDCl3 (400 MHz). (F) NOESY NMR spectrum of OALC in CDCl3 (400 MHz). (PDF) [file pone.0132791.s008.pdf]

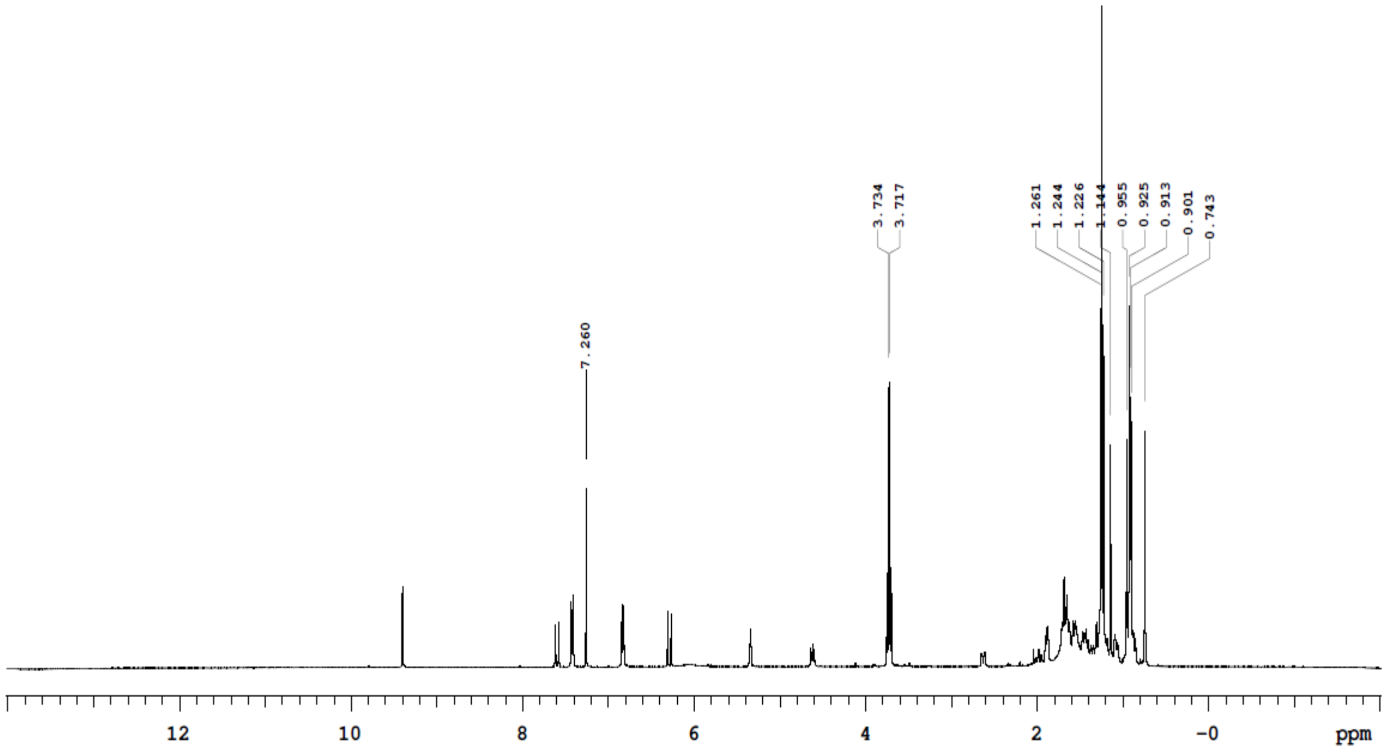

Figure S3A. ^{1}H NMR spectrum of oleanolic aldehyde coumarate in $CDCl_3$ (400 MHz)

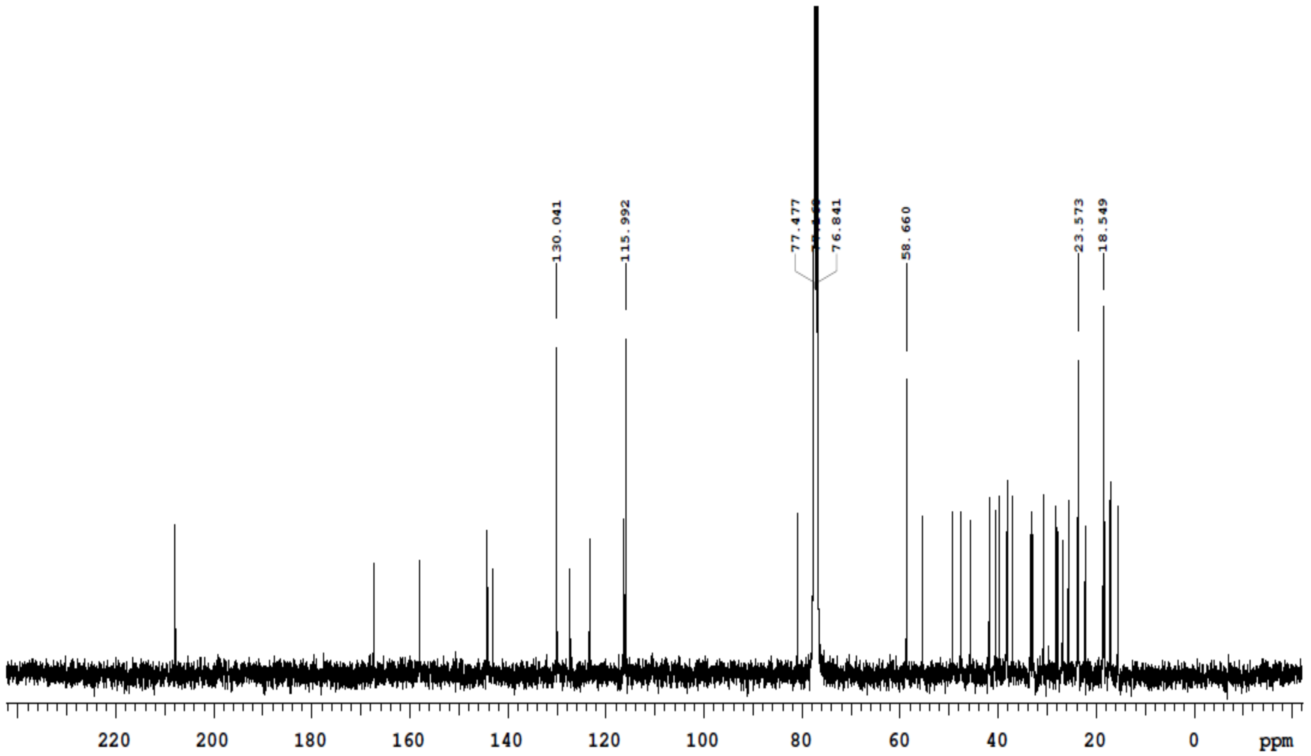

Figure S3B. ^{13}C NMR spectrum of oleanolic aldehyde coumarate in $CDCl_3$ (400 MHz)

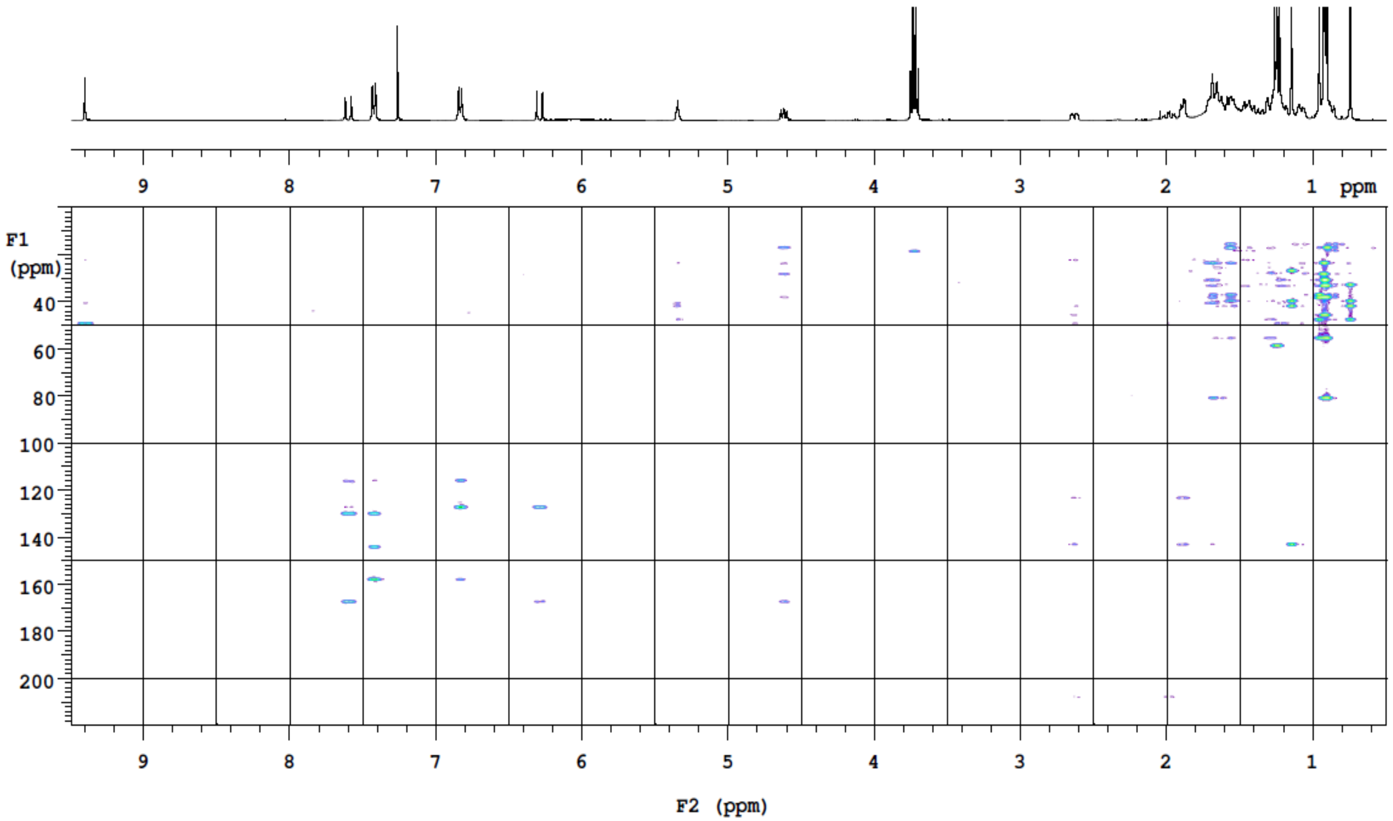

Figure S3C. HSQC NMR spectrum of oleanolic aldehyde coumarate in $CDCl_3$ (400 MHz)

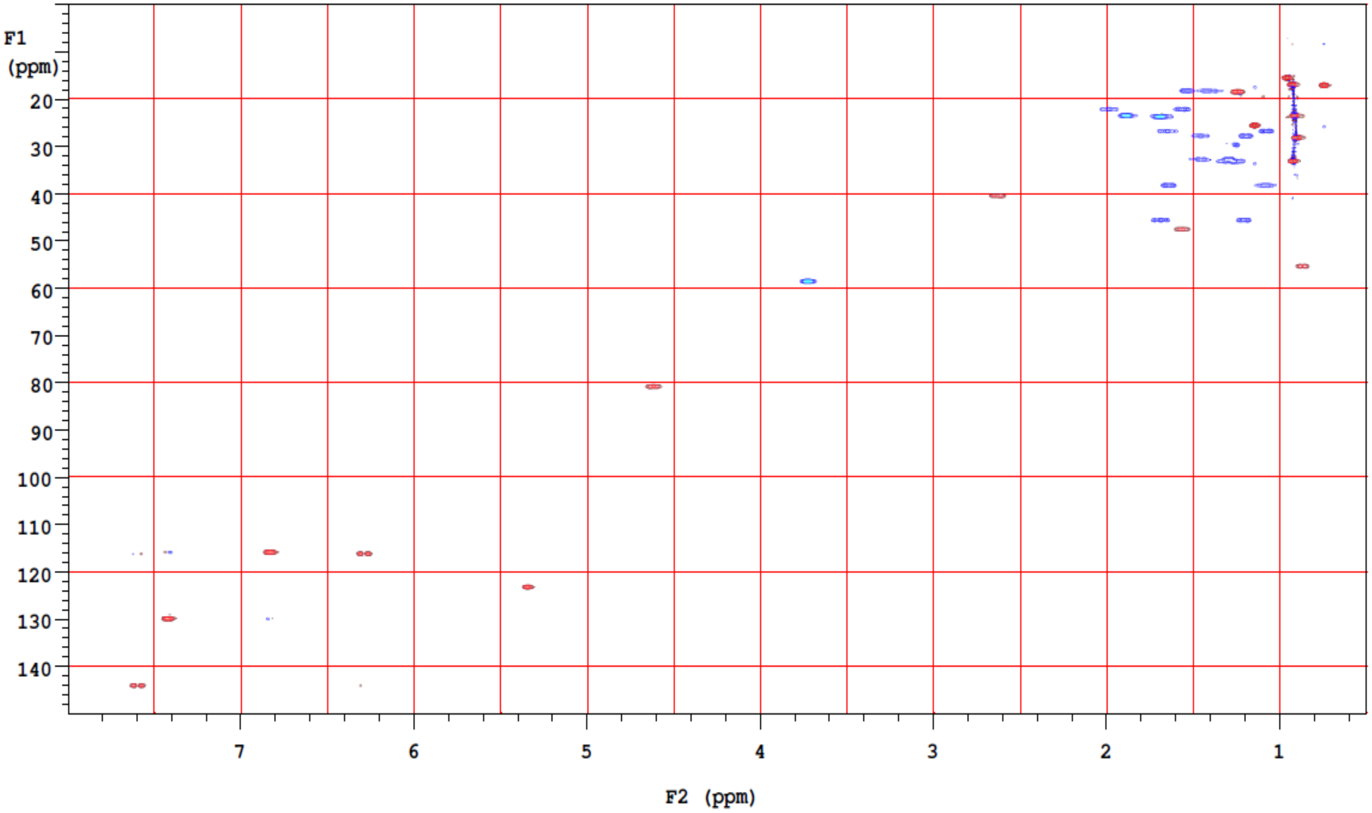

Figure S3D. HMBC NMR spectrum of oleanolic aldehyde coumarate in $CDCl_3$ (400 MHz)

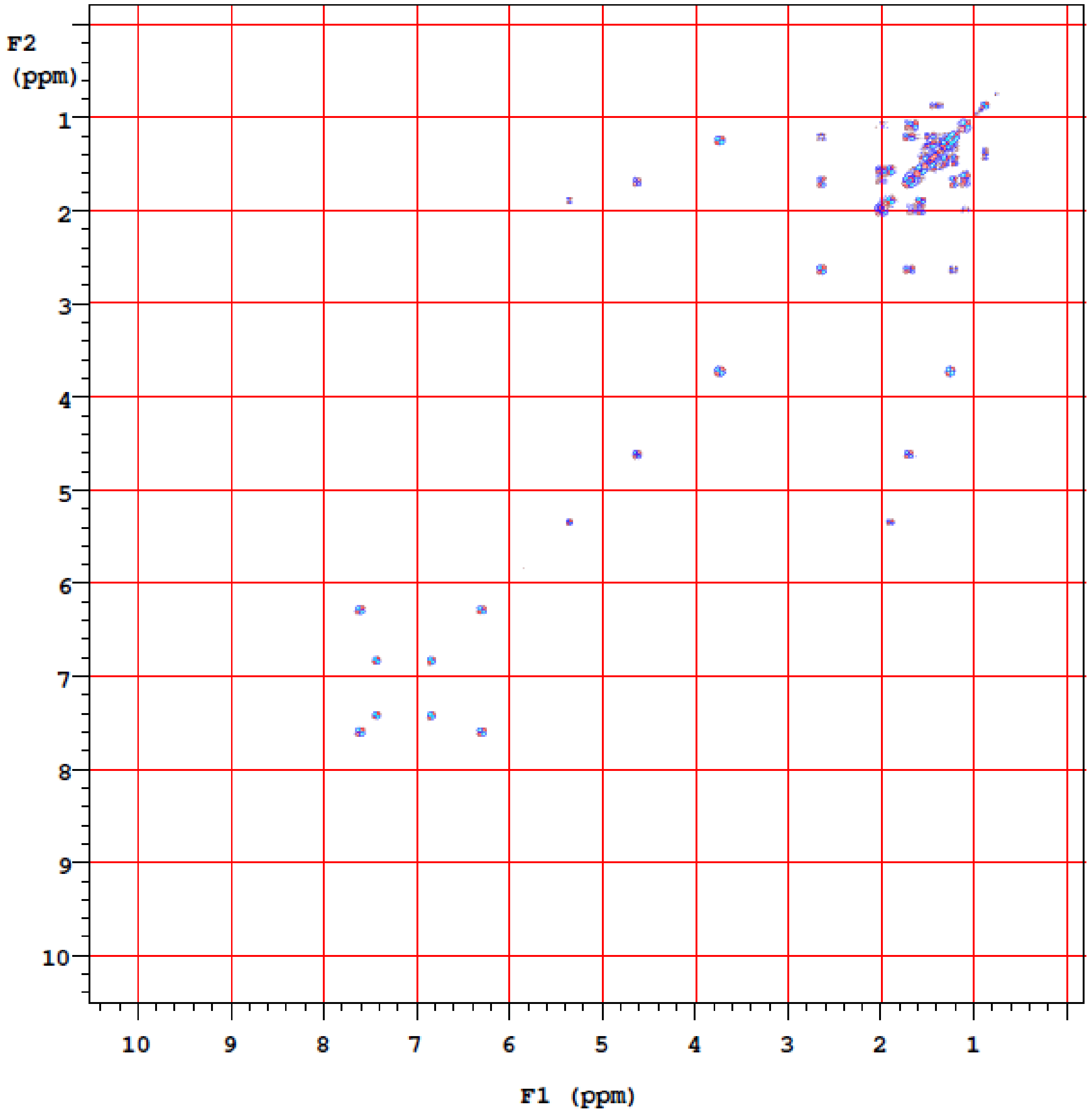

Figure S3E. COSY NMR spectrum of oleanolic aldehyde coumarate in $CDCl_3$ (400 MHz)

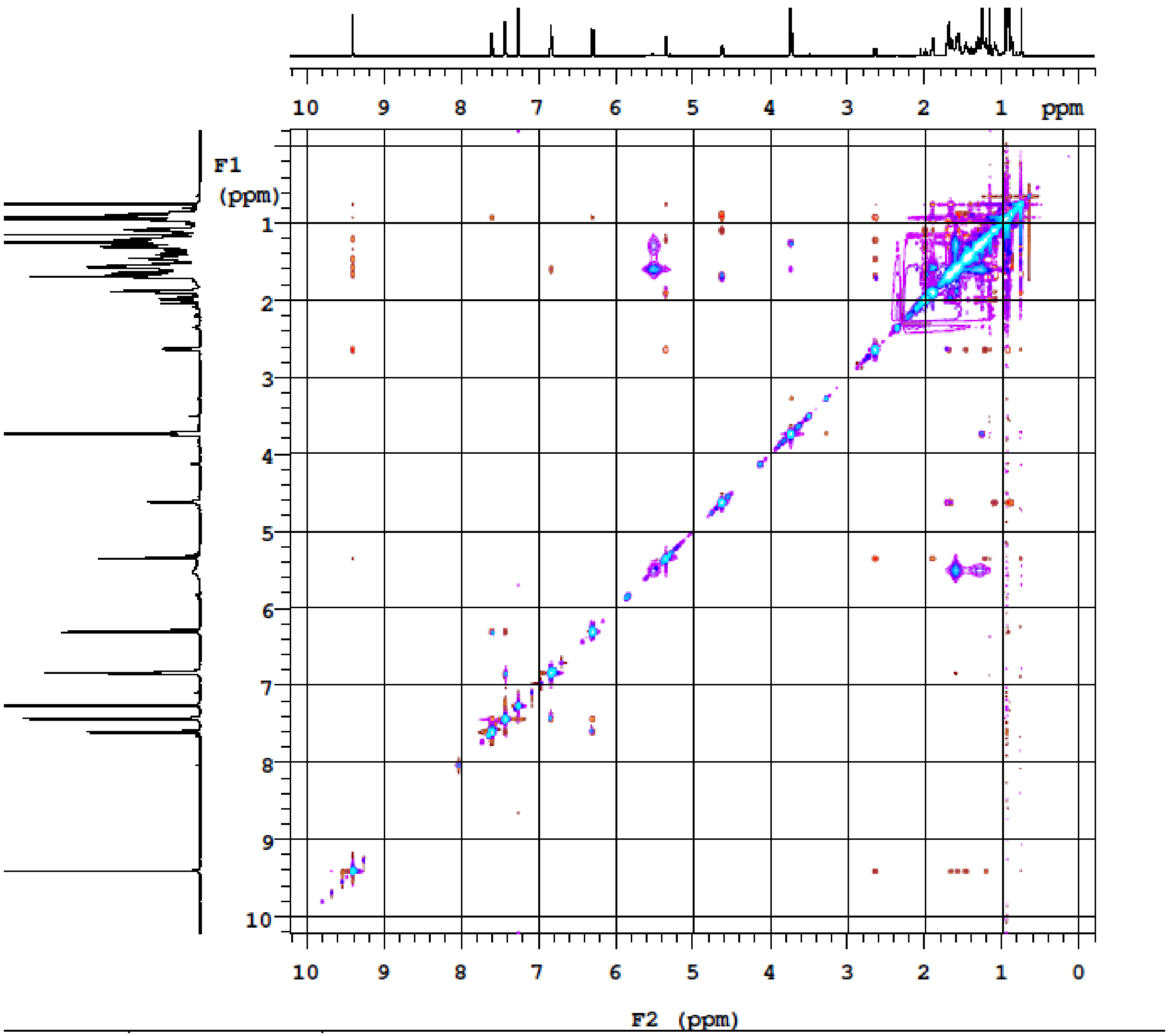

Figure S3F. NOESY NMR spectrum of oleanolic aldehyde coumarate in $CDCl_3$ (400 MHz)
